# Identification of *Postn*^+^ periosteal progenitor cells with bone regenerative potential

**DOI:** 10.1172/jci.insight.182524

**Published:** 2024-10-08

**Authors:** Bei Yin, Fangyuan Shen, Qingge Ma, Yongcheng Liu, Xianglong Han, Xuyu Cai, Yu Shi, Ling Ye

**Affiliations:** 1 State Key Laboratory of Oral Diseases,; 2 National Center for Stomatology,; 3 National Clinical Research Center for Oral Diseases, West China Hospital of Stomatology,; 4 Department of Endodontics, West China School of Stomatology,; 5 Department of Thoracic Surgery, West China Hospital,; 6 Department of Orthodontics, West China School of Stomatology, and; 7 Institute of Respiratory Health, Frontiers Science Center for Disease-related Molecular Network, West China Hospital, Sichuan University, Chengdu, China.

**Keywords:** Bone biology, Adult stem cells, Bone marrow

## Abstract

Bone contains multiple pools of skeletal stem/progenitor cells (SSPCs), and SSPCs in periosteal compartments are known to exhibit higher regenerative potential than those in BM and endosteal compartments. However, the in vivo identity and hierarchical relationships of periosteal SSPCs (P-SSPCs) remain unclear due to a lack of reliable markers to distinguish BM SSPCs and P-SSPCs. Here, we found that periosteal mesenchymal progenitor cells (P-MPs) in periosteum can be identified based on *Postn*-CreER^T2^ expression. *Postn*-expressing periosteal subpopulation produces osteolineage descendants that fuel bones to maintain homeostasis and support regeneration. Notably, *Postn*^+^ P-MPs are likely derived from *Gli1*^+^ skeletal stem cells (SSCs). Ablation of *Postn*^+^ cells results in impairments in homeostatic cortical bone architecture and defects in fracture repair. Genetic deletion of *Igf1r* in *Postn*^+^ cells dampens bone fracture healing. In summary, our study provides a mechanistic understanding of bone regeneration through the regulation of region-specific *Postn*^+^ P-MPs.

## Abstract 2

Postn is a reliable marker to distinguish bone marrow mesenchymal progenitors and periosteal mesenchymal progenitors. Postn+ periosteal mesenchymal progenitors maintain homeostasis and support regeneration.

## Graphical abstract

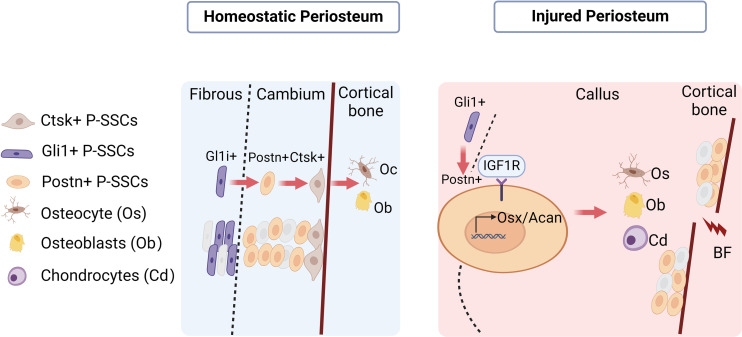

## Introduction

The skeleton contains multiple distinct skeletal stem/progenitor cell (SSPC) pools with diverse functional properties. The efficient isolation of SSPCs can provide a therapeutic cell source for skeletal injury. The challenges in such an approach lie in characterizing SSPCs and identifying specific markers to distinguish their origin (1, 2). Extensive studies have established that SSPCs are highly enriched in the BM compartment (3); however, studies of periosteal SSPCs are limited. The periosteum is a membrane lining the surface of bone and is divided anatomically into a fibrous layer (outer membrane) and cambium layer (inner membrane) according to its morphological and regional characteristics. The periosteum exerts key roles in bone regeneration by supplying SSPCs (4–6). Notably, periosteal SSPCs reportedly exhibit greater regenerative potential than BM stem cells (BM-SSPCs), potentially resulting from greater clonogenicity, migration, and differentiation capacity (7, 8). Despite the critical role of periosteum-based healing, studies on P-SSPCs are limited due to the challenge of identifying reliable genetic markers specific to the periosteum.

The periosteal markers known at present are inadequately specific. For example, *Ctsk-Cre* labels P-SSPCs in the periosteum but is also a well-known marker of osteoclasts (9). *Sca1* (10), *Pdgfr*α (11), *Prx1* (12–14), *Sox9* (15, 16), *aSMA* (17, 18), *Nestin* (19), and *Lepr* (20) are identifiable not only in periosteal cells but also in a substantial number of endosteal and BM cells (21). Therefore, markers to specifically distinguish P-SSPCs from BM-SSPCs are needed.

*Gli1^+^* cells were previously reported to label mesenchymal stem cells in calvarial sutures and regulate bone development and regeneration (22). We previously demonstrated that early postnatal *Gli1*^+^ skeletal stem cells (SSCs) in the chondro-osseous junction promote bone growth and repair (23). Consistent with these findings, a recent study showed that *Gli1*^+^ SSCs in the periosteum are primarily in charge of the bone regeneration in bicortical but not stabilized fractures (24). Despite these findings, *Gli1-CreER^T2^* recombines in only a few cells within the diaphysis of femurs, suggesting that certain *Gli1*^–^ periosteal cells also contribute to bone fracture healing, potentially through distinct properties from those of *Gli1*^+^ SSCs.

Emerging evidence shows that periostin (Postn), an extracellular matrix (ECM) protein present in the periosteum and in the periodontal ligament, controls self-renewal ability of periosteal mesenchymal progenitor cells (P-MPs) and plays a key role in bone biology. The serum level of POSTN is associated with the porosity of cortical bone (25). Loss of the *Postn* gene in mice leads to skeletal abnormalities (26, 27). Importantly, *Postn* contributes to periosteal activation and stem cell niche maintenance after skeletal injury. Knocking out *Postn* in mice compromises bone healing, leading to fibrosis and nonunion (7). Despite the central function of *Postn* gene in bone healing and despite it being widely used as a gene marker of periosteum/cambium layer (28), the in vivo cytological characteristics and physiological specializations of *Postn*^+^ cells in the skeleton remain elusive.

The heterogeneity underlying periosteal cells of bone callus has been reported. Single-cell RNA-Seq (scRNA-Seq) of the periosteum of uninjured tibias and day 3, day 5, day 7 callus identified populations of SSPCs, fibrogenic cells, osteoblasts, chondrocytes, etc., and found that SSPCs expressing *Ly6a* were at the apex of differentiation (29). The study concentrated on the early/inflammatory/hematoma stage of bone healing. The heterogeneity of mesenchymal cells in the fibrocartilaginous/remodeling stage remains elusive.

Here, scRNA-Seq and genetic lineage-tracing experiments revealed a subset of P-MPs expressing *Postn*. Loss of *Postn*^+^ cells damages cortical bone formation under steady-state conditions and bone regeneration during defect healing. Intriguingly, adult *Postn*^+^ P-MPs may originate from *Gli1*^+^ P-SSCs in response to injury. Mechanistically, we demonstrate that IGF1 signaling was indispensable for *Postn*^+^ P-MPs function in bone repair. Together, these findings provide insights into P-MPs identification and their role in bicortical fracture healing regulation.

## Results

### Identification of Postn^+^ P-MPs utilizing scRNA transcriptome profiling.

**Figure 1:**
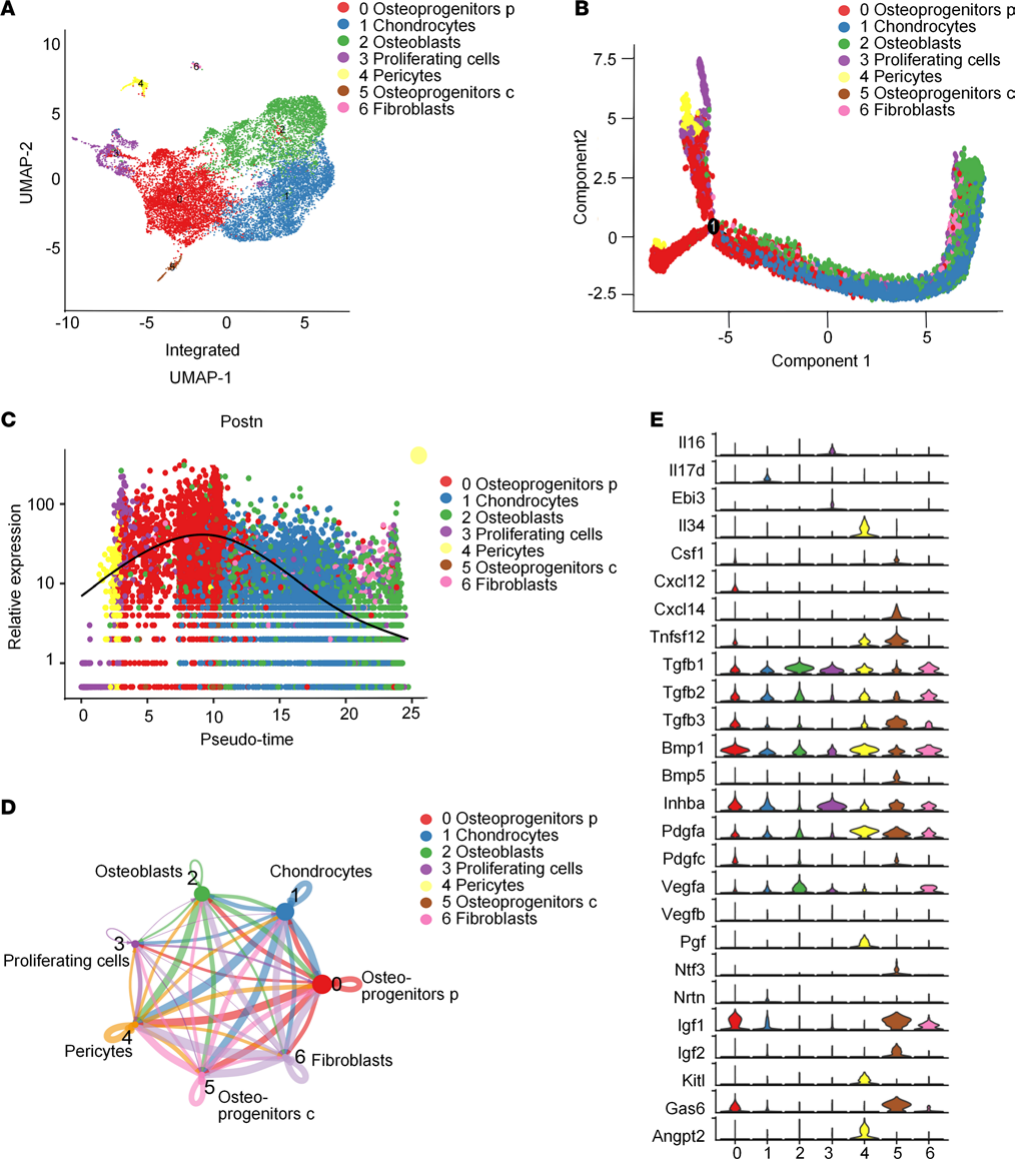
Identification of *Postn*^+^ P-MPs utilizing scRNA transcriptome profiling. (**A**) UMAP revealed cellular heterogeneity of mesenchymal cells in bone callus with 7 distinct clusters by integrating day 7, day 10, and day 14 data set. (**B**) Pseudotime ordering of callus mesenchymal cells by pseudotime Monocle trajectory plot. (**C**) *Postn* expression in each cell along the pseudotime axis. (**D**) The cellular interaction among distinct clusters based on CellChat analysis. (**E**) Representative growth factors and cytokines in each cluster. See also Supplemental Figure 1.

To define all the periosteal populations present in bone callus of the fibrocartilaginous stage, we performed no-bias scRNA-Seq of nonhematopoietic mesenchymal cells (CD45^–^CD31^–^TER119^–^) in day 7, day 10, and day 14 callus. Mesenchymal stromal cells comprising the callus were clustered into 7 groups (Figure 1A) with their representative gene expression signatures shown in a heatmap (Supplemental Figure 1A; supplemental material available online with this article; https://doi.org/10.1172/jci.insight.182524DS1). Cluster 0 was defined as *Postn*^+^ osteoprogenitors (osteoprogenitors p) with the representative genes *Aspn*, *Ogn*, *Ptn*, and *Postn*. *Aspn* and *Ogn* are reportedly present in osteoprogenitor cells in the developing limbs (30). *Ptn* is known to mark *Cxcl12*-abundant reticular cells rather than mature osteoblast cells in BM (31). *Postn* is reportedly expressed in periosteum and contributes to skeletal regeneration (7). Other clusters included chondrogenic cells (expressing *Cnmd* and *Ucma*), osteogenic cells (expressing *Bglap* and *Ibsp*), proliferating cells (expressing *Mki67* and *Ccnb2*), *Ctsk*^+^ osteoprogenitors (osteoprogenitors c) (expressing *Prrx1* and *Ctsk*), fibroblasts (expressing *S100a4* and *CCN2*), and pericytes (expressing *Rgs5* and *Mcam*), which were similar to the day 10 callus data reported by Ambrosi et al. (32).

The pseudotime trajectory appeared in a Y shape with 2 branches indicating direct intramembranous ossification and endochondral ossification (Figure 1B). *Postn^+^* cluster demonstrated an enriched distribution at the starting point of the differentiation route (Figure 1B and Supplemental Figure 1B), suggesting that the *Postn*^+^ cluster is likely the ancestor of other mesenchymal cells in the data set. The heatmap revealed genes differentially expressed in a manner dependent on the pseudotime line, and *Postn* was among the genes potentially responsible for the intramembranous ossification routes (Supplemental Figure 1C). Notably, *Postn* expression peaked in *Postn*^+^ osteoprogenitors and decreased in osteoblasts and chondrocytes during differentiation (Figure 1C). Consistently, *Postn*^+^ cells are distributed in the late mesenchymal progenitor cluster according to the data set (NCBI’s Gene Expression Omnibus [GEO] database, GSE108892) of single-cell transcriptomics of femoral mesenchymal cells in ref. 29 (Supplemental Figure 1D).

CellChat analysis revealed that robust interaction occurred among divergent clusters, and *Postn*^+^ cluster and chondrocytes had the most active interaction (Figure 1D). Growth factors and cytokines facilitate the communication among divergent cells, playing central roles in initiating and mediating the injury-induced responses (33, 34). *Postn*^+^ cluster, chondrocytes, and osteoblasts possessed the largest number of growth factors and cytokines (Supplemental Figure 1E). Among these cytokines, *Igf1* and *BMP1* are highly expressed by *Postn*^+^ cluster (Figure 1E), indicating the key function of these signals in the *Postn^+^* cluster^.^

### Postn marks long-term skeletal progenitor cells within the periosteum.

**Figure 2:**
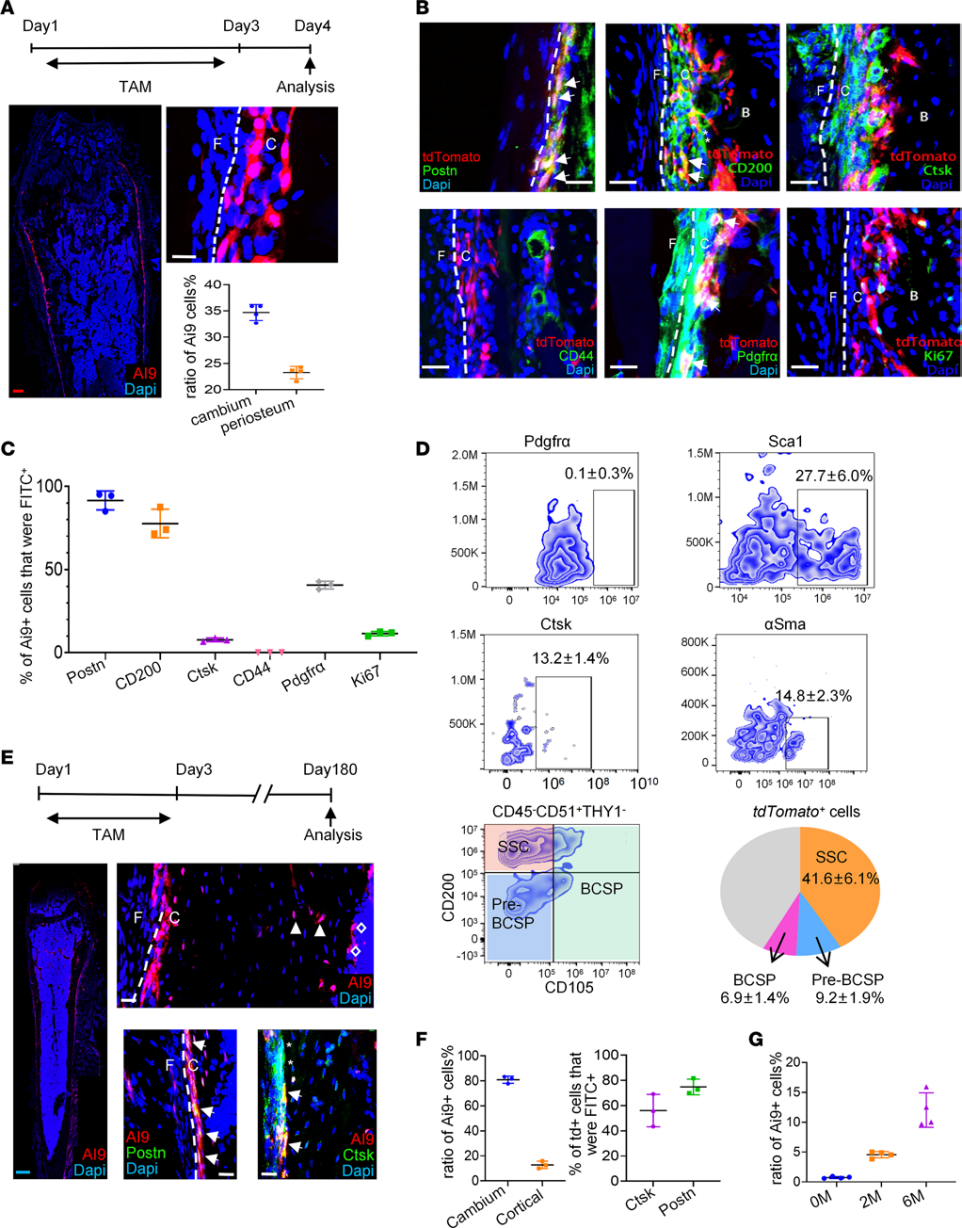
The *Postn* reporter activity labeled long-time (more than 6 months) retaining periosteal stem cells. (**A**) The *Postn* reporter activity was shown in femur of *Postn-CreER^T2^; tdTomato* mice with tamoxifen injected for consecutive 3 days (*n* = 4). The graph shows the quantitative statistics of the ratio of *tdTomato* in cambium and periosteum. (**B**) Immunofluorescence staining of select MSCs markers and Ki67 in the metaphyseal femur of *Postn-CreER^T2^; tdTomato* mice (*n* = 3). The arrow indicates the colocalization, and the asterisk indicates the single staining of target protein. (**C**) The graph was the quantitative percentage of *tdTomato^+^* cells that were immunofluorescence positive. (**D**) Flow cytometry analysis of select MSCs markers in *tdTomato^+^* periosteal cells (*n* = 3) (tamoxifen injected for consecutive 3 days and sample acquired on fourth day). Pie chart illustrates the percentage of BCSP, Pre-BCSP, and SSC in *tdTomato^+^* cells. (**E**) Immunofluorescence staining of POSTN and CTSK in femur of *Postn-CreER^T2^; tdTomato* mice 6 months after tamoxifen injection (*n* = 3). The arrowheads indicate the *tdTomato* signal in cortical bone, the diamonds indicate the *tdTomato* signal in endosteum, the arrows indicate the colocalization, and the asterisks indicate the single staining of target protein. (**F**) The left graph indicated the ratio of *tdTomato^+^* cells in cambium layer and cortical bone osteocytes. The right graph indicated the percentage of *tdTomato*^+^ cells that were CTSK^+^ or POSTN^+^. (**G**) Ratio of *tdTomato^+^* cells among cortical bone osteocytes after tracing for 0, 2, and 6 months (*n* = 4 for each group). Data were obtained from 3 independent experiments. Data are presented as mean ± SD. Red scale bar: 200 μm. Blue scale bar: 500 μm. White scale bar: 20 μm. F, fibrous layer; C, cambium layer; B, cortical bone.

We marked *Postn*^+^ cells using a transgenic approach in which the expression of the *tdTomato* reporter was controlled by tamoxifen-inducible *Cre* (*Postn-CreER^T2^; tdTomato*). Leakage of *CreER* was barely observed in the absence of tamoxifen (Supplemental Figure 2A). After tamoxifen administration for 3 consecutive days, 2-month-old transgenic mice developed a population of labeled cells predominantly present in the inner cambium layer of the femur. *Postn* reporter activity in adult mice accounted for 34.7% ± 1.5% of the cells in the inner cambium layer, 23.1% ± 1.3% of the cells in the entire periosteum (Figure 2A). Minimal (0.9% ± 0.2%) osteocytes in cortical bone were also labeled by *tdTomato* (Supplemental Figure 2B). Despite an overwhelming preference in periosteum, 10.0% ± 0.8% *tdTomato*^+^ cells were also present in the BM compartment, especially in the trabecular bone surface (Supplemental Figure 2B). Apart from femoral periosteum, *Postn* reporter activity also marked the periodontal membrane, most of the cells in the periosteum of vertebrae, ribs and cranium, a small portion of cells in BM, and endosteum of vertebrae, ribs, and cranium (Supplemental Figure 2C). The distribution of *Postn*^+^ cells highlighted the presence of a cell population preferentially residing in the cambium of periosteum.

Putative MP markers were evaluated by flow cytometry and immunofluorescence staining after tamoxifen injection for 3 consecutive days to study the features of *Postn*^+^ cells. As expected, almost all of the *tdTomato*^+^ cells were copositive for POSTN per immunofluorescence staining (Figure 2, B and C). CD200 was widely expressed in the inner cambium layer and represented 73.0% ± 8.6% of the entire spectrum of *Postn*^+^ cells (Figure 2, B and C). Although both *Ctsk*^+^ and *Postn*^+^ cells were located in the inner cambium layer of the periosteum, *Postn^+^* cells were more closely associated with cortical bone, and there was minor colocalization with *Ctsk*^+^ cells (7.8% ± 1.1%) (Figure 2, B and C). Consistent with the general presence of CD44^+^ cells in the BM, the *tdTomato*^+^ status and CD44 expression status were mutually exclusive (Figure 2, B and C). *Pdgfr*α^+^ cells were present in both the cambium layer and the fibrous layer, and they showed overlapping with 40.8% ± 2.3% *Postn*^+^ cells (Figure 2, B and C). In addition, 21.6% ± 1.3% *tdTomato*^+^ cells in cambium labeled osteoblasts, as revealed by OSX staining (Supplemental Figure 2D). Importantly, Ki67 staining was observed in a small fraction (11.5% ± 1.3%) of *Postn*^+^ cells, almost equal to that of *Postn^–^* cells in cambium (10.7% ± 1.3%), indicating that *Postn^+^* cells are slowly proliferating under steady-state conditions (Figure 2, B and C). This is consistent with a recent report that cambium layer periosteal cells (CL-PCs) are proliferating while fibrous layer periosteal cells (FL-PCs) are dormant (28). FACS analysis revealed that *tdTomato*^+^ cells expressed select MP markers, such as SCA1 (27.7% ± 6.0%) and αSMA (14.8% ± 2.3%) (Figure 2D and Supplemental Figure 2E). Consistent with immunofluorescence staining, a small proportion of *tdTomato*^+^ cells expressed CTSK (13.2% ± 1.4%), but they barely expressed PDGFRα. Using the marker combinations reported by Chan et al. (35), approximately half of the *tdTomato*^+^ cells were identified as SSCs (CD45^–^CD51^+^CD90^–^Ly51^–^CD105^–^CD200^+^) (41.6% ± 6.1%), a much smaller percentage was identified as pre–bone cartilage and stromal progenitor (pre-BCSP) (CD45^–^CD51^+^THY1^–^Ly51^–^CD105^–^CD200^–^) (9.2% ± 1.9%), and BCSP (CD45^–^CD51^+^THY1^–^Ly51^–^CD105^+^) (6.9% ± 1.4%) (Figure 2D). The *tdTomato*^+^ cells were chased for either 2 or 6 months to determine whether *Postn*^+^ cells were long-lived MPs. Following these long chasing periods, *tdTomato*^+^ cells were distributed in various bone compartments, including periosteum, cortical bone (12.0% ± 2.9% of the osteocytes), BM, and endosteum (Figure 2, E and F, and Supplemental Figure 2F). In the periosteum, *tdTomato*^+^ cells were exclusively present in the cambium with an increased prevalence (reaching as high as 81.0% ± 2.9%) (Figure 2, E and F). Notably, most of the *Postn*^+^ cells and their progenies in the periosteum maintained the cellular identities during a tracing period. They maintained POSTN expression (74.8% ± 6.2%) (Figure 2, E and F) and showed major overlapping with CD200, while they separated from CD44 and CD34 (CD34 was located in the fibrous layer) (Supplemental Figure 2F). In addition, *Postn*^+^ cells were of low proliferation capability in homeostasis (Supplemental Figure 2F). Unexpectedly, a prominently greater percentage of *tdTomato*^+^ (56.1% ± 12.9%) cells expressed CTSK after long tracing than at the initial labeling, suggesting that *Postn*^+^ cells can contribute to the formation of *Ctsk*^+^ cells during homeostasis (Figure 2, E and F). Particularly, adult *Postn*^+^ cells consistently made increasingly larger contributions to cortical osteocytes over time (Figure 2G). These data indicate that adult *Postn^+^* cells, as P-MPs, are long-term periosteum residency cells*,* highly express CD200, and give rise to osteocytes during cortical bone homeostasis.

### Preexisting Postn^+^ MPs are responsible for cortical bone formation after fracture.

**Figure 3:**
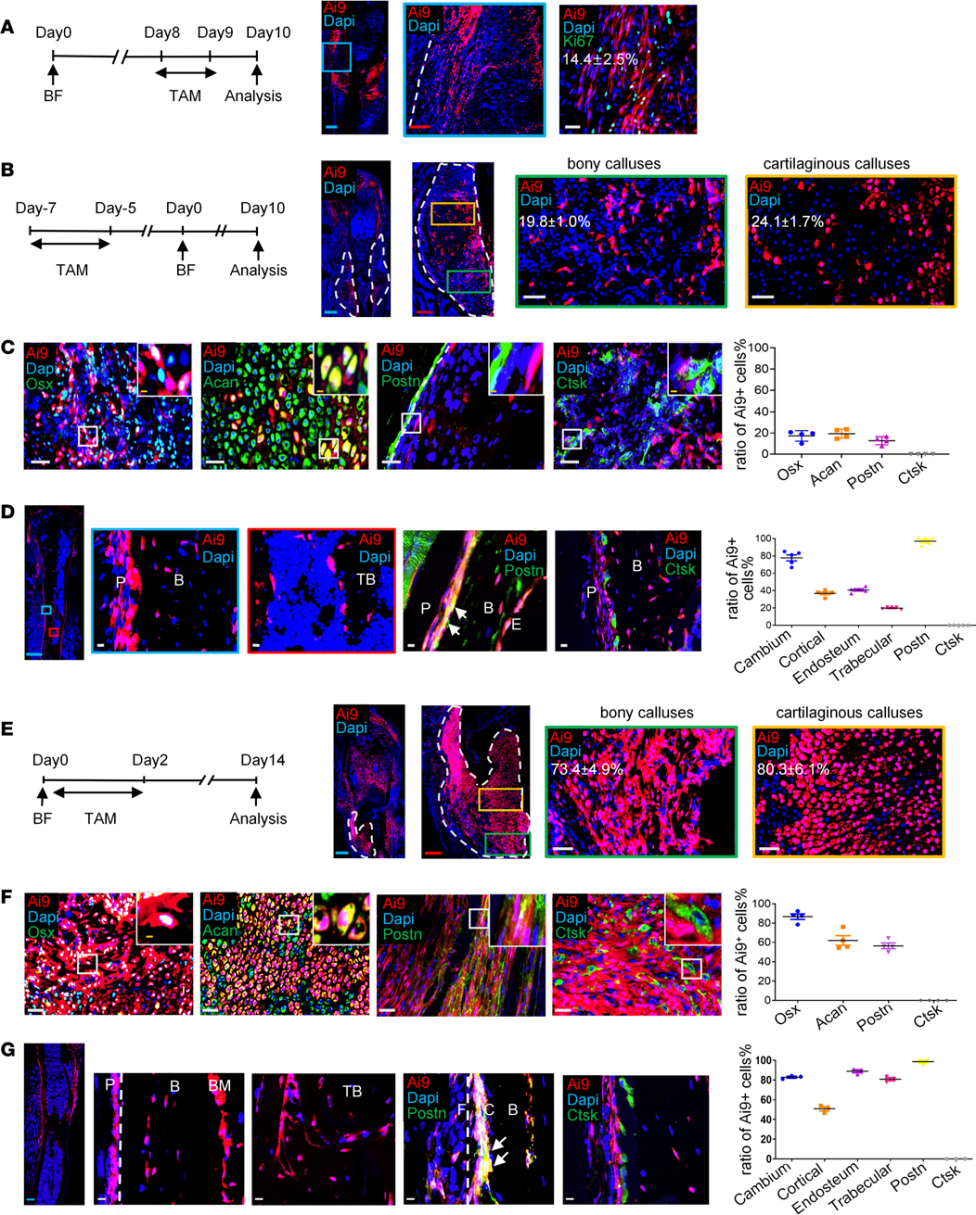
*Postn* reporter activity marked periosteal cells that contributed to bone healing. (**A**) Distribution of *Postn* reporter activity and Ki67 immunofluorescence staining in day 10 callus of *Postn-CreER^T2^; tdTomato* mice with tamoxifen injection at postfracture days 8 and 9 (*n* = 3). (**B** and **C**) Ratio of *dTomato^+^* cells in hard and soft callus and immunofluorescence staining of OSX, ACAN, POSTN, and CTSK in day 10 callus with tamoxifen injection 8 days before bone fracture for consecutive 3 days (*n* = 4). The graph indicates percentage of *tdTomato^+^* cells. (**D**) Distribution of *tdTomato* signal and immunofluorescence staining of POSTN and CTSK 2 months after fracture with tamoxifen injection 8 days before the bone fracture (*n* = 5). The graph indicated the ratio of *tdTomato^+^* cells in cambium, cortical bone, endosteum, trabecular bone, POSTN-expressing cells, and CTSK-expressing cells. (**E** and **F**) Ratio of *tdTomato^+^* cells in hard and soft callus and immunofluorescence staining of OSX, ACAN, POSTN, and CTSK in day 10 callus with tamoxifen injection for 3 days starting from the fracture day (*n* = 4). The graph indicated percentage of *tdTomato^+^* cells. (**G**) Distribution of *tdTomato^+^* cells and immunofluorescence staining of POSTN and CTSK 2 months after fracture with tamoxifen injection starting at the fracture day for 3 days (*n* = 3). The graph indicates the ratio of *tdTomato^+^* cells in cambium, cortical bone, endosteum, trabecular bone, POSTN-expressing, and CTSK-expressing cells. Data were obtained from 3 independent experiments. Data are presented as mean ± SD. Blue scale bar: 500 μm. Red scale bar: 200 μm. The arrow indicates the colocalization. White scale bar: 20 μm. Orange scale bar: 5 μm. F, fibrous layer; C, cambium layer; B, cortical bone; E, endosteum; P, periosteum.

We next conducted further studies of *Postn*^+^ P-MPs in response to injury. *Postn*-*CreER^T2^*; *tdTomato* mice were injected with tamoxifen twice at day 8 and day 9 after operation, and samples were acquired at day 10. It indicated that *Postn* reporter activity was mainly located in the outer membranous region of the callus and the thickening periosteum close to the cortical bone, rather than in the central hard callus (Figure 3A). *Postn*^+^ cells were of moderate proliferative capability (14.4% ± 2.5%) (Figure 3A). For day 3 and day 5 callus, *Postn*^+^ cells were predominantly enriched in the thickening periosteum (Supplemental Figure 3A), and their percentage peaked at day 5 among the above 3 time points (Supplemental Figure 3B). To study the progeny of *Postn*^+^ P-MPs, tamoxifen was administered up to 8 days before fracture for 3 consecutive days to mark the preexisting *Postn*^+^ P-MPs (pre-*Postn*^+^ P-MPs). At 3, 5, and 7 days after fracture, the *tdTomato*^+^ cells were frequently enriched in the thickening periosteum (Supplemental Figure 3, C–E). At 10 days after fracture, *tdTomato*^+^ cells representing the pre-*Postn*^+^ P-MPs and their progenies appeared mainly in the callus and periosteum, and only a small number of *tdTomato*^+^ cells existed in the BM. Reporter activity corresponded to 19.8% ± 1.0% of the bony calluses (Figure 3B) and 24.1% ± 1.7% of the cartilaginous calluses at day 10 (Figure 3B). Specifically, *tdTomato*^+^ cells contributed to 17.4% ± 5.0% of the osteoblasts marked by OSX and 19.4% ± 4.4% of the chondrocytes marked by ACAN (Figure 3C). While some *tdTomato^+^* cells were positive for POSTN in thickening periosteum (12.8% ± 3.8%), they separated from each other in the peripheral region of callus (Figure 3C). There was little overlap with the expression of CTSK (Figure 3C). Notably, preexisting *Postn*^+^ cells changed their proliferation status in response to injury. As reflected by Ki67 staining, the proliferation rate of *Postn* lineage (*tdTomato*^+^) cells was high (23.1% ± 0.9%) in day 3 callus (Supplemental Figure 3C), but it dropped to 10.6% ± 2.4% in day 10 calluses (Supplemental Figure 3F), similar to *Postn*^+^ cells in homeostasis (11.5% ± 1.3%) (Figure 2, B and C). At 2 months after fracture, when bone healing was almost complete, the *tdTomato*^+^ cells not only constituted 36.9% ± 3.6% of the osteocytes in the cortical bone, 40.8% ± 3.0% of the endosteal cells (Figure 3D), and 19.8% ± 1.2% of the osteocytes in the newly formed trabecular bone (Figure 3D), but they also recovered their residency at the periosteum (accounting for 77.8% ± 7.9% of the cells in the cambium and 97.0% ± 4.3% POSTN-expressing periosteal cells; Figure 3D). Notably, tdTomato^+^ cells hardly contributed to *Ctsk^+^* periosteal cells (Figure 3D). Taken together, these findings suggest that pre-*Postn*^+^ P-MPs and their progeny contribute to bone formation during bone fracture healing.

### Postn^+^ P-MPs labeled upon bone injury make greater contributions to bone regeneration.

*TdTomato*^+^ cells were clearly integrated into the central region of the callus, and the *Postn*^+^ cells in the peripheral region of the callus were *tdTomato*^–^ (Supplemental Figure 3G). Thus, we hypothesized that fresh *Postn*^+^ P-MPs emerged in response to injury. Tamoxifen was administered from the operation day to 2 days after fracture to test this hypothesis. The distribution of P-MPs expressing *Postn* and their progenies labeled around the fracture day (post-*Postn*^+^ P-MPs) differed extensively from the preexisting ones (pre-*Postn*^+^ P-MPs). Post-*Postn*^+^ P-MPs and their progenies persistently occupied a prominently larger proportion of the day 3, day 7, and day 10 calluses than pre-*Postn*^+^ P-MPs did (Supplemental Figure 3H), and they unexpectedly emerged in the BM compartment, particularly below the growth plate (Figure 3E). *Td-Tomato^+^* cells in BM may derive from a small quantity of preexisting Postn^+^ cells and SSCs in the osteo-chondro-progenitor zone. As quantified, 73.4% ± 4.9% of the cellular components in day 10 bony calluses (Figure 3E) and 80.3% ± 6.1% of the cellular components (Figure 3E) in cartilaginous calluses were derived from post-*Postn*^+^ P-MPs. Subsequent immunofluorescence staining revealed that most of the OSX^+^ cells (86.7% ± 5.9%) and ACAN^+^ chondrocytes (61.9% ± 10.0%) were also *tdTomato*^+^ (Figure 3F). Notably, the progenies of post-*Postn*^+^ P-MPs expressed a remarkably greater proportion of POSTN than those derived from pre-*Postn*^+^ P-MPs (56.6% ± 7.2% versus 12.8% ± 3.8%) (Figure 3, C and F). Similarly, progenies of *td-Tomato*^+^ cells labeled upon injury showed a much larger overlapping with Pdgfrα- or CD200-expressing cells than those of preexisting *Postn*^+^ P-MPs (Supplemental Figure 3, I–K). Few *tdTomato*^+^ cells coexpressed CTSK (Figure 3F). Consistent with the immunofluorescence staining results, FACS analysis revealed that select *tdTomato*^+^ cells expressed PDGFRα (18.7% ± 3.1%), SCA1 (48% ± 4.6%), and CD200 (54.3% ± 6.0%) (Supplemental Figure 2G). These findings indicate that, upon injury, the newly generated *Postn*^+^ P-MPs contribute greatly to replenishing the periosteum of the callus. In terms of proliferative capability, the post-*Postn*^+^ P-MPs (15.4% ± 2.8%) were similar to the pre-*Postn*^+^ P-MPs (Supplemental Figure 3L). Two months after a bone fracture, the *tdTomato*^+^ cells contributed to 50.8% ± 3.7% of the cortical osteocytes, 88.9% ± 2.8% of the endosteal cells (Figure 3G), and 80.8% ± 2.8% of the osteocytes in the newly formed trabecular bone (Figure 3G) and 82.8% ± 1.7% of the cambium (specifically 98.6% ± 2.4% of the POSTN^+^ cells; Figure 3G). *TdTomato*^+^ cells seldom overlapped with CTSK expression at the month-2 time point (Figure 3G). Taken together, these findings suggest that post-*Postn*^+^ P-MPs are major cell sources involved in biocortical bone fracture healing.

In addition to bicortical fractures, post-*Postn*^+^ P-MPs also contributed to drill injury repair (Supplemental Figure 4A). Different from the long bones, the calvarial bone only goes through intramembranous ossification during bone formation. We thus further confirmed the contribution of *Postn*^+^ P-MPs to the intramembranous bone formation by establishing a model of calvarial defects (Supplemental Figure 4B).

### Gli1^+^ cells gave rise to Postn^+^ cells in response to bone injury.

**Figure 4:**
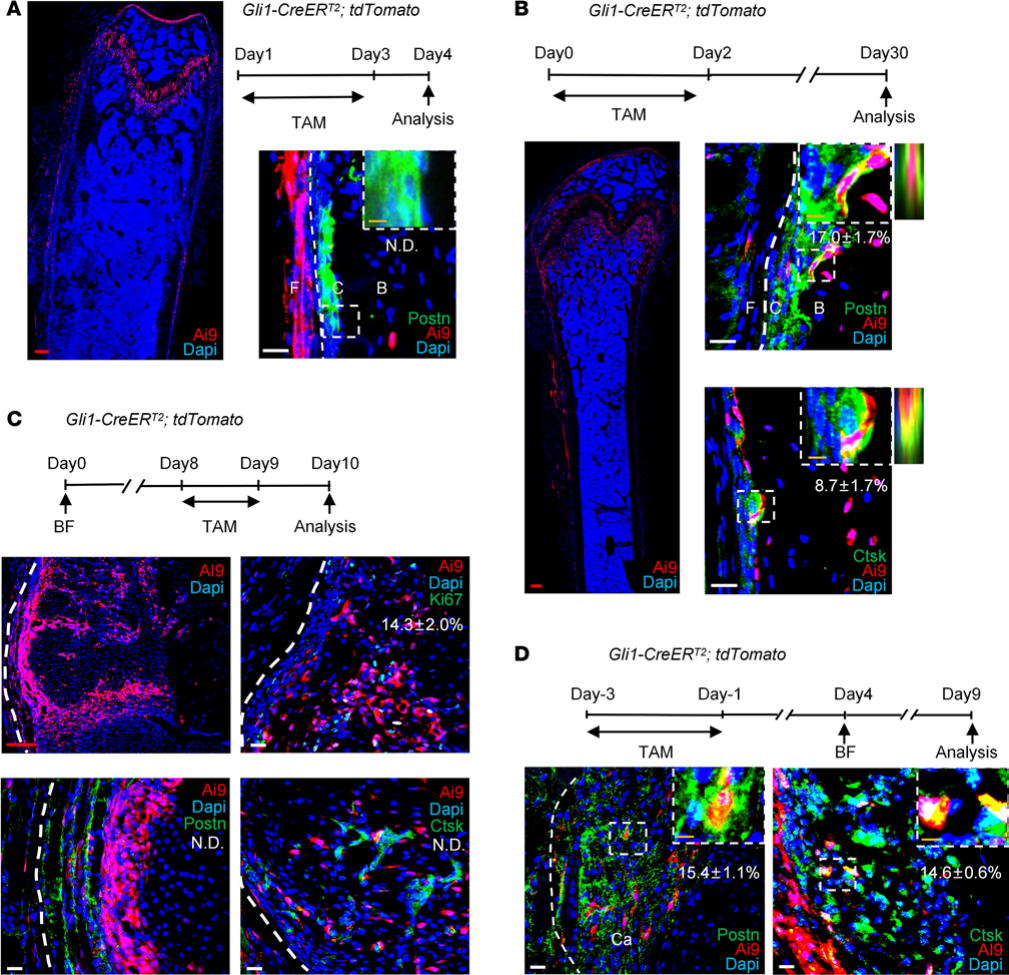
The *Postn*^+^ cells may be derived from *Gli1*^+^ cells. (**A**) The distribution of *Gli1* reporter activity and immunofluorescence staining of POSTN in the femoral metaphyseal periosteum of *Gli1-CreER^T2^; tdTomato* mice with tamoxifen injection for 3 days and samples acquired at day 4. (**B**) The distribution of *tdTomato* reporter activity and immunofluorescence staining of POSTN and CTSK in *Gli1-CreER^T2^; tdTomato* mice 1 month after tamoxifen injection for 3 days. To the right of the dashed box showed *Z* stack images of the cell in dashed box. (**C**) The distribution of *tdTomato* reporter activity and immunofluorescence staining of KI67, POSTN, and CTSK in day 10 callus of *Gli1*-*CreER^T2^*; *tdTomato* mice with tamoxifen injection at post fracture days 8 and 9. (**D**) The immunofluorescence staining of POSTN and CTSK in day 5 callus of *Gli1*-*CreER^T2^*; *tdTomato* mice. Data were obtained from 3 independent experiments. Data are presented as mean ± SD. *n* = 3 per genotype. Red scale bar: 200 μm. White scale bar: 20 μm. Orange scale bar: 5 μm. F, fibrous layer; C, cambium layer; B, cortical bone; Ca, callus. See also Supplemental Figure 4.

Post-*Postn*^+^ P-MPs appeared to make a dramatically greater contribution to fracture healing than pre-*Postn*^+^ P-MPs, suggesting that other populations may acquire *Postn* expression in the initial period of bone injury. The fibrous layer is considered a stem cell source that supplements the cambium and the cortical bone. We then explored whether the fibrous population *Gli1*^+^ P-SSCs gave rise to *Postn*^+^ P-MPs. One-month-old *Gli1-CreER^T2^; tdTomato* mice were administered with tamoxifen for 3 consecutive days. *Gli1*^+^ P-SSCs marked by *tdTomato* were located in the fibrous layer and separated from *Postn*^+^ P-MPs (Figure 4A). After tracing *Gli1^+^* periosteal cells for 1 month, they migrated inward and became *Postn*^+^ and *Ctsk*^+^ cells (Figure 4B). Therefore, in homeostasis, at least part of the *Postn*^+^ and *Ctsk*^+^ cells were derived from fibrous *Gli1*^+^ P-SSCs. To demonstrate the expression of *Gli1* in day 10 callus, tamoxifen was administered at day 8 and day 9 after fracture. *Gli1^+^* cells was mainly enriched in the outer membrane of day 10 callus (Figure 4C) with moderate proliferating capability (14.3% ± 2.0%) (Figure 4C), and they seldom showed overlapping with POSTN and CTSK expression (Figure 4C). We then explored whether *Gli1^+^* P-SSCs generated *Postn*^+^ P-MPs during bone healing. The *Gli1-CreER^T2^; tdTomato* mice were administered tamoxifen 8 days before bone fracture. *Gli1*^+^ cells overlapped partially with *Postn*^+^ cells in day 5 (15.4% ± 1.1%); this overlapping largely disappeared in day 10 callus (Figure 4D and Supplemental Figure 4C). Interestingly, *Gli1*^+^ cells consistently contributed to *Ctsk^+^* cells in day 5 and day 10 callus (Figure 4D and Supplemental Figure 4D). We then evaluated *Gli1* expression via in situ hybridization (ISH) in *Postn-creER^T2^; tdTomato* mice. There were few *Gli1^+^* P-SSCs in the callus, and there was no costaining with *Postn*-lineage cells (Supplemental Figure 4E). These findings suggest that *Gli1*^+^ P-SSCs are early in the hierarchy of periosteal cell derivatives upon bicortical fracture, and their decedents, such as *Postn*^+^ P-MPs and *Ctsk*^+^ P-MPs, are functional populations that contribute to bone healing.

### Postn^+^ cells can be reactivated in successive bone injuries.

**Figure 5:**
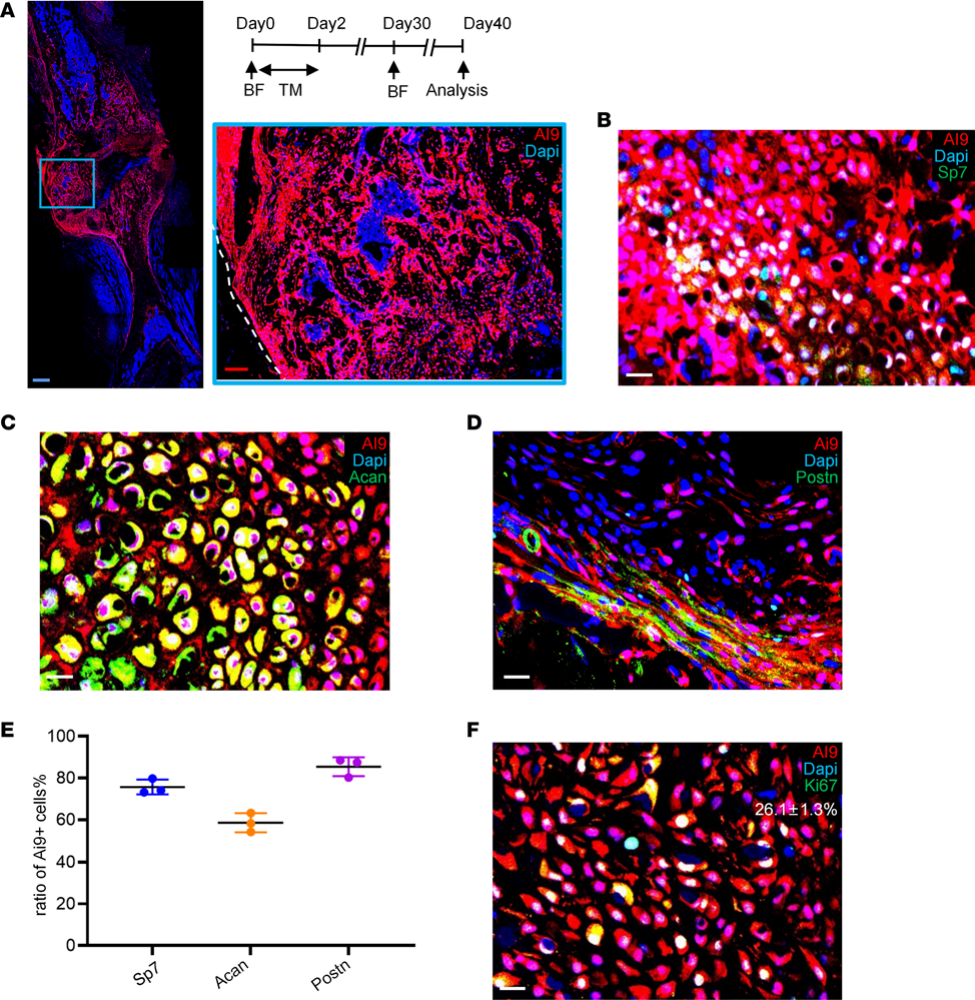
*Postn^+^* cells could be reactivated in a successive bone fracture. (**A**–**D**) Distribution of *tdTomato^+^* cells (**A**) and immunofluorescence staining of OSX (Sp7) (**B**), ACAN (**C**), and POSTN (**D**) in day 10 callus of second fracture in *Postn*-*CreER^T2^; tdTomato* mice. Tamoxifen was injected for 3 consecutive days starting from the fracture day. A second fracture was performed 1 month after first one, and day 10 callus of the second fracture was examined. (**E**) The graph showed the ratio of *tdTomato^+^* cells in POSTN^+^, OSX^+^, and ACAN^+^ cells. (**F**) Immunofluorescence staining of Ki67 in day 10 callus of second fracture in *Postn-CreERT2; tdTomato* mice. Data were obtained from 3 independent experiments. Data are presented as mean ± SD. *n* = 3 per genotype. Blue scale bar: 500 μm. Red scale bar: 200 μm. White scale bar: 20 μm. F, fibrous layer; C, cambium layer; B, cortical bone; Ca, callus. See also Supplemental Figure 5.

We then investigated whether the repopulated *tdTomato*^+^ cells could contribute to the regeneration of a recurring fracture in the same bone. The second fracture was performed 1 month after the first one, when *tdTomato*^+^ cells comprised 40.3% ± 4.7% and 49.8% ± 5.3% of periosteal cells for preexisting (“pre” strategy) or injury-induced (“post” strategy), respectively (Supplemental Figure 5, A–C), which is a larger percentage compared with that in homeostasis (23.1% ± 1.3%) (Figure 2A). Besides, *tdTomato* also labeled cortical osteocytes and trabecular bone osteoblasts (Supplemental Figure 5, A–C). Therefore, *tdTomato-*labeled above populations may make larger contributions in the second fracture compared with the first one. Our results reveal that the progenies of post-*Postn*^+^ P-MPs/*tdTomato*^+^ cells largely constituted the callus (Figure 5A) and specifically contributed to 75.7% ± 3.5% of the OSX^+^ osteoblasts (Figure 5, B and E) and 58.6% ± 4.6% of the ACAN^+^ chondrocytes (Figure 5, C and E). The majority of the *Postn*^+^ cells (Figure 5, D and E) (85.5% ± 4.5%) were *tdTomato*^+^, which was identical to the contribution of post-*Postn*^+^ P-MPs, confirming our hypothesis that *Gli1*^+^ cells differentiated into *Postn*^+^ P-MPs and replenished the *Postn*^+^ P-MPs population. Therefore, post-*Postn*^+^ P-MPs persist in the new periosteum after the completion of fracture repair and are reactivated to repair successive bone injury. Consistent with the contribution of *Postn*^+^ P-MPs to the first-round fracture, the reestablished *tdTomato*^+^ cells began to proliferate; 26.1% ± 1.3% of these cells were Ki67^+^ (Figure 5F), confirming the presence of self-renewing osteochondroprogenitors. Consistently, the pre-*Postn*^+^ P-MPs reconstituted the callus in the second fracture (Supplemental Figure 5D).

### Depletion of periosteal Postn^+^ cells impaired cortical bone homeostasis.

**Figure 6:**
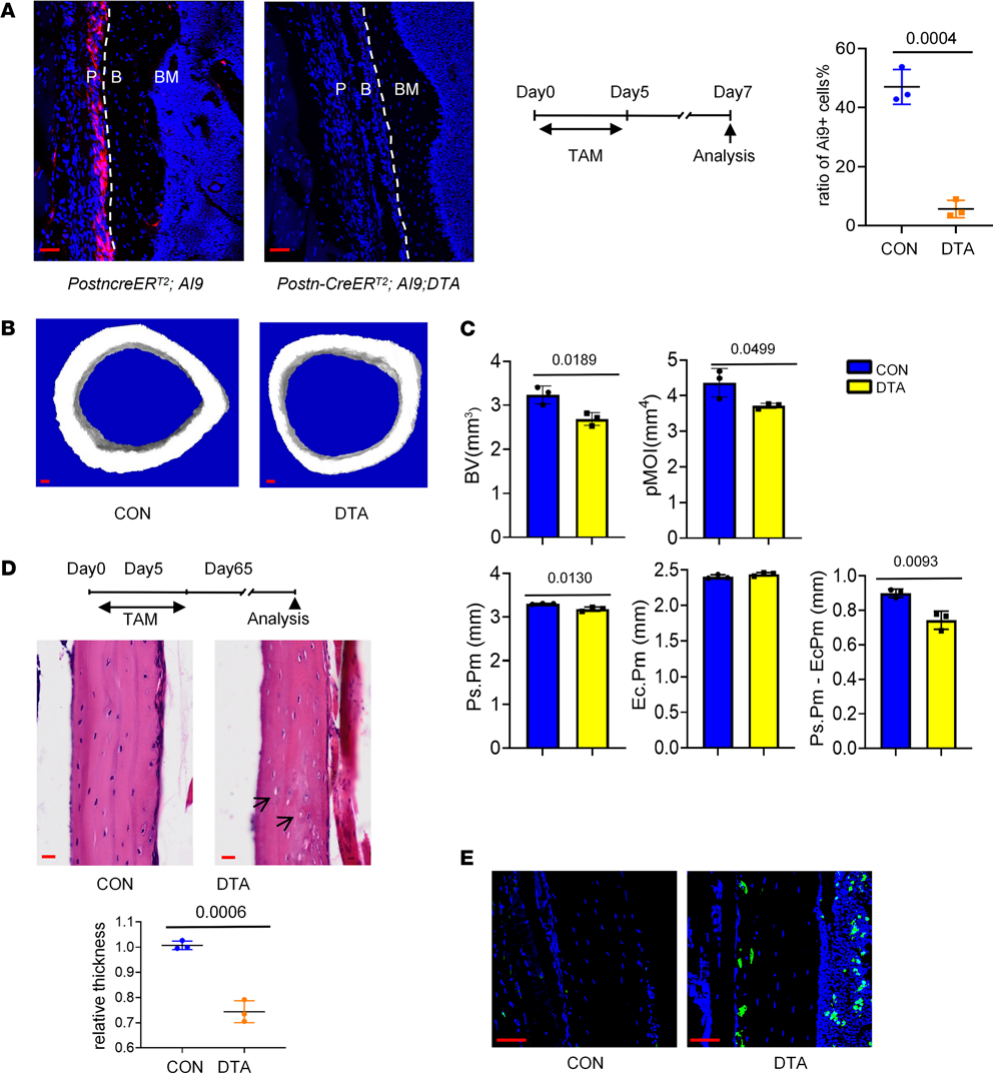
The ablation of *Postn*^+^ cells impaired cortical bone formation in homeostasis. (**A**) Distribution of *tdTomato^+^* cells in periosteum of *Postn-CreER^T2^; tdTomato* (CON) mice and *Postn-CreER^T2^; tdTomato; DTA* (DTA) mice. Tamoxifen was injected for 5 days, and samples were acquired at seventh day. B, cortical bone; P, periosteum. (**B**) μ-CT reconstructions of midshaft femoral cortical bone. (**C**) The cortical bone parameter of midshaft femoral cortical bone by CT analysis in the *Postn-CreER^T2^; DTA* mice and control mice (*Postn-CreER^T2^*). The analysis included the bone volume (B.V), polar moment of inertia (pMOI), outer/periosteal perimeter (Ps.Pm), endocortical perimeter (Ec.Pm), and Ps.Pm versus Ec.Pm. (**D**) The H&E staining of femoral bone in the *Postn-CreER^T2^; DTA* mice and control mice. The graph showed the relative thickness of midshaft cortical bone. (**E**) Tunel staining in CON and DTA group. Data were obtained from 3 independent experiments. Statistics, standard 2-tailed Student’s *t* test. Data are presented as mean ± SD. *n* = 3 per genotype. Red scale bar: 50 μm. The arrows indicate the vacuolar fossa in the cortical bone.

To determine the significance of the *Postn*^+^ P-MPs, *Postn-CreER^T2^; Rosa26-DTA* transgenic mice (abbreviated as DTA mice) were established, in which the *Postn*^+^ P-MPs were specifically depleted when tamoxifen-induced diphtheria toxin was expressed. In the DTA mice injected with tamoxifen, the percentage of *Postn*^+^ cells largely decreased in response to tamoxifen injection, as reflected by the loss of *tdTomato*^+^ cells (Figure 6A). The cortex was strongly affected 2 months after tamoxifen administration under steady-state conditions, as reflected by the decreased bone volume, polar moment of inertia, periosteal perimeter (Ps.Pm) and Ps.Pm versus endocortical perimeter (Ec.Pm) observed via μ-CT analysis (Figure 6, B and C) and decreased thickness of the cortical bone according to H&E staining (Figure 6D). Notably, the nuclei were also missing or shrunk, and more apoptotic cells were observed upon *Postn*^+^ P-SSC depletion (Figure 6E). The underlying mechanisms may lie in 2 aspects. On one hand, tamoxifen administration directly killed the minimal osteocytes labeled by *tdTomato* (Supplemental Figure 2B). On the other hand, *Postn*^+^ periosteal cells may affect osteocytes in a paracrine manner through osteocyte dendritic processes, which was similar with the case that osteocytic RANKL was transmitted to osteoclast precursors through osteocyte dendritic processes that extended beyond the bone surface into BM and periosteum (36). Thus, *Postn*^+^ P-MPs are indispensable for establishing periosteal bone formation and maintaining cortical bone homeostasis.

### Ablation of periosteal Postn^+^ cells impaired bone healing.

**Figure 7:**
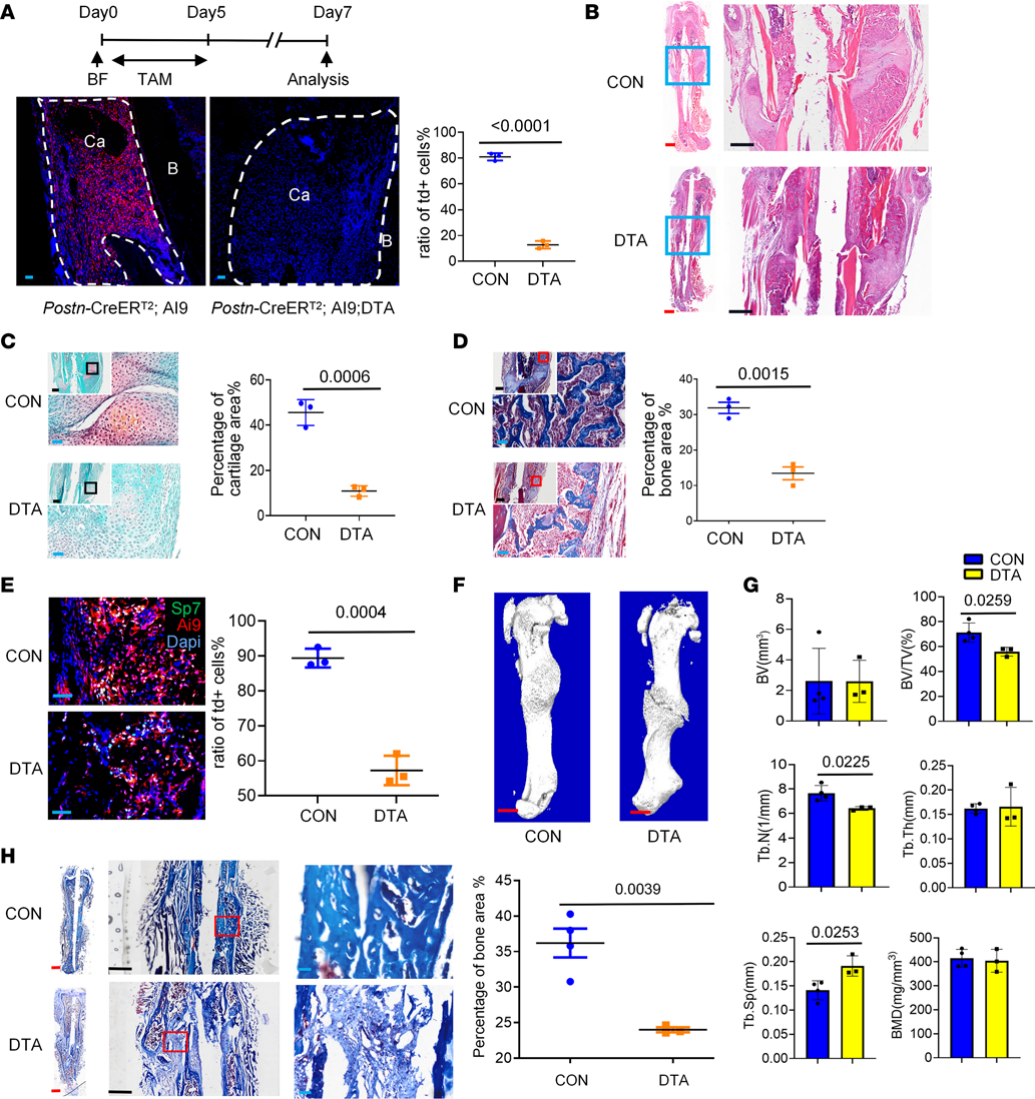
The deletion of *Postn^+^* cells impaired bone healing. (**A**) Distribution of *tdTomato^+^* cells in day 7 callus of *Postn-CreER^T2^; tdTomato* (CON) and *Postn-CreER^T2^; tdTomato; DTA* (DTA) mice with 5 day tamoxifen administration starting from the fracture day. B, cortical bone; Ca, callus (*n* = 3 per genotype). (**B**–**D**) The H&E staining (**B**), safranin O (**C**), and Masson’s trichrome staining (**D**) of day 14 callus in DTA mice and control mice (*n* = 3 per genotype). The graph illustrates the percentage of cartilage area (**C**) and bone area (**D**). (**E**) The immunofluorescence staining of OSX (Sp7) in day 14 callus in the *Postn-CreER^T2^; tdTomato; DTA* mice and control mice (*n* = 3 per genotype). The graph illustrates the ratio of *tdTomato^+^* in OSX-expressing cells. (**F** and **G**) The μCT reconstructions (**F**) and comparative parameters (**G**) of the callus 1 month post fracture (*n* = 4 in CON group and *n* = 3 in DTA group). The analysis included the bone volume (B.V), bone volume/tissue volume (BV/TV), trabecular number (Tb.N), trabecular thickness (Tb.Th), trabecular separation (Tb.Sp), and bone mineral density (BMD). (**H**) The Masson’s trichrome staining of the fractured bone in DTA and control mice 1 month after fracture (*n* = 4 in CON group and *n* = 3 in DTA group). Data were obtained from 3 independent experiments. Standard 2-tailed Student’s *t* test. Data are presented as mean ± SD. Red scale bar: 1 mm. Black scale bar: 500 μm. Blue scale bar: 50 μm.

We then administered tamoxifen at the time of bone injury to explore the importance of *Postn*^+^ P-MPs in bone regeneration of adult mice. Almost no *tdTomato*^+^ cells were observed in the DTA mice after administration of tamoxifen for 5 consecutive days, demonstrating the success of *Postn*^+^ P-SSC ablation (Figure 7A). Histological staining of the day 14 callus indicated that the proportion of the bone area and cartilage area was decreased in the ablation group (Figure 7, B–D). Moreover, the contribution of *tdTomato*^+^ cells to osteoblasts was decreased in the DTA mice, as indicated by OSX immunofluorescence staining (Figure 7E). One-month after fracture, μ-CT analysis showed that the ablation of *Postn*^+^ P-MPs led to a striking reduction in the bone volume percentage, a decrease in trabecular bone number, and an increase in trabecular bone separation (Figure 7, F and G). In addition, the DTA mice exhibited nonunion of fibrous tissue rather than mature mineralized tissue filling the broken ends (Figure 7H). Taken together, these findings suggest that functional *Postn*^+^ P-MPs are indispensable for driving bone regeneration after bone fracture.

### Conditional deletion of Igf1r in Postn^+^ cells compromised bone healing.

**Figure 8:**
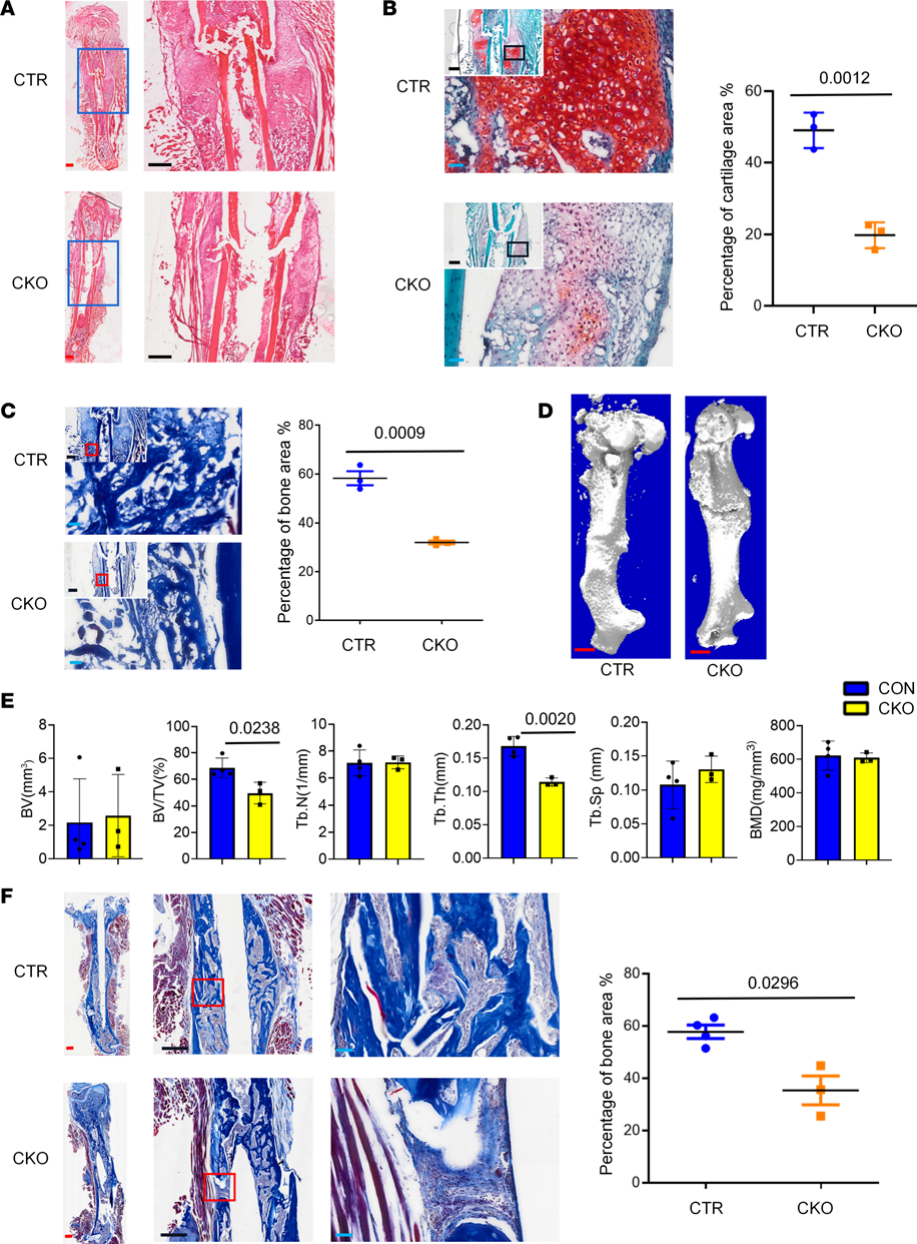
CKO of *Igf1r* in *Postn*^+^ cells compromised bone healing. (**A**–**C**) The H&E staining (**A**), safranin O (**B**), and Masson’s trichrome staining (**C**) of the day 14 callus in the *Postn-CreER^T2^; Igf1r^fl/fl^* mice (CKO) and control mice (*Igf1r^fl/fl^*) (*n* = 3). The graph illustrates percentage of cartilage area (**B**) and bone area (**C**). (**D** and **E**) The μ-CT reconstructions (**D**) and analytical results (**E**) in the callus 1 month after fracture (*n* = 4 in CTR group and *n* = 3 in CKO group). The analysis included the bone volume (B.V), bone volume/tissue volume (BV/TV), trabecular number (Tb.N), trabecular thickness (Tb.Th), trabecular separation (Tb.Sp), and bone mineral density (BMD). (**F**) The Masson’s trichrome staining of the callus 1 month after fracture (*n* = 4 in CTR group and *n* = 3 in CKO group). Data were obtained from 3 independent experiments. Standard 2-tailed Student’s *t* test. Data are presented as mean ± SD. Red scale bar: 1 mm. Black scale bar: 500 μm. Blue scale bar: 50 μm.

Next, we sought to investigate the regulatory mechanisms of *Postn*^+^ P-MPs in bicortical bone fracture healing. Given the putative enrichment of IGF signal in the *Postn*^+^ cluster, we speculated that IGF1 signal held tremendous significance in the functional specialization of *Postn*^+^ cells. Therefore, we conditionally knocked out *Igf1r* in *Postn*^+^ cells in *Postn-CreER^T2^; Igf1r^fl/fl^* mice. Tamoxifen injection started 5 days before the fracture day. The conditional KO (CKO) mice exhibited impaired fracture healing. In detail, the percentage of bone and cartilage area was compromised at 14 days after fracture (Figure 8, A–C). The bone volume percentage was largely decreased 1 month after fracture according to the μ-CT analysis (Figure 8, D and E). Nevertheless, the quality of new bone was not substantially altered, aside from a decrease in thickness. Masson’s trichrome staining confirmed that the nonunion site was fibrous tissue (Figure 8F). It is highly plausible that *Postn*^+^ cells depend on the coordination of multiple-signal pathway such as BMP and Gas6 signaling, which are also highly expressed by *Postn*^+^ cells. Overall, the *Postn*^+^ P-MPs retained their functionality at least partially through IGF1 signaling.

## Discussion

The periosteum contains multiple SSPCs pools that manage cortical bone homeostasis and fracture repair. The in vivo origin and regulatory mechanism of SSPCs at distinct bone compartments have long been controversial due to their substantial heterogeneity and lack of specific markers. Here, by no-bias scRNA-Seq, we identified a population of *Postn*^+^ P-MPs possessing the features of MPs that are critical for cortical bone formation and repair. Spatially, *Gli1*^+^, *Lepr*^+^, and *Pdgfr*α^+^ periosteal cells are located in the fibrous layer (11), while *Ctsk*^+^, *Prx1*^+^, *Mx1*^+^*aSma*^+^, *Col2*^+^, *Osx*^+^, and *Postn*^+^ periosteal cells are located in the cambium of the periosteum during homeostasis (7, 28). *Postn*^+^ P-MPs predominantly coexpressed CD200 rather than CTSK initially but continuously generated *Ctsk*^+^ cells over time under steady-state conditions. However, during bicortical fracture, descendants of both pre- and post-*Postn*^+^ P-MPs barely expressed CTSK, suggesting that injury may stimulate a distinct population of persistent *Postn*^+^ cells. This heterogeneity of *Postn*^+^ P-MPs requires further investigation.

One of the notable differences between post-*Postn*^+^ P-MPs and pre-*Postn*^+^ P-MPs is that the progenies of post-*Postn*^+^ P-MPs reestablish the periosteum of the callus during healing, while those of the pre-*Postn*^+^ P-MPs do not. It is possible that, upon fracture, other P-SSCs differentiate into *Postn*^+^ cells and replenish the pool of P-MPs. *Gli1*^+^ cells are considered SSCs in multiple tissues (37). Our previous studies demonstrate that *Gli1* generally marks metaphyseal mesenchymal SSCs and plays an indispensable role in bone formation (23). Located in the fibrous layer of the periosteum, *Gli1*^+^ SSCs potentially supplement *Postn*^+^ cells in the cambium layer. We identified a hierarchical relationship between *Gli1*^+^, *Postn*^+^, and *Ctsk*^+^ cells in terms of homeostasis and repair. It is speculated that *Gli1*^+^ P-SSCs in both the periosteum and the metaphyseal compartment may acquire *Postn* expression early in bone healing. This may help explain the substantially greater contribution of injury-induced *Postn*^+^ cells versus that of preexisting cells. Moreover, *Postn* expression in progenies of *Gli1*^+^ cells may be activated directly by injury or indirectly through crosstalk between P-MPs and the metaphyseal compartment, similar to the regulation of growth plate resting zone stem cells by P-SSC–derived Indian hedgehog (IHH) (38). The exact upstream and downstream relationships between *Gli1*^+^ P-SSCs and *Postn*^+^ P-MPs can be explored further by more advanced genetic approaches, such as interleaved reporter transgenic mice (39, 40). Quite different from the cellular hierarchical relationships between fibrous layer and cambium layer, Liu et al. (28) recently found PCs from the 2 layers are in charge of distinct biological events. CL-PCs contribute to homeostatic bone formation, while FL-PCs mainly participate in bone injury healing. Nevertheless, they did notice a contribution of CL-PCs in early callus (day 2 and day 4) while day 14 callus was overwhelmingly composed of FL-PCs and their progenies (28). Contradictorily, previous studies reported that both CL-PCs and FL-PCs — including Mx1^+^αSmα^+^ (17), CTSK^+^ (9), LepR^+^ (41), CD51^+^ (42), αSMA^+^ (18), and Prx1^+^ (43) periosteal cells in cambium layers, and Gli^+^ (24) and Sca1^+^ (42) cells in fibrous layer — have participated in bone injury healing. In addition, Pdgfrα^+^ FL-PCs are reported to participate in cortical bone homeostasis and bone fracture healing (11), similar to *Postn*^+^ CL-PCs. It is highly plausible that a hierarchical relationship exists between the cambium layer and fibrous layer and that both layers contribute to cortical bone homeostasis and fracture healing. For example, PDGFRα^+^ FL-PCs give rise to Nestin^+^ CL-PCs, and *Gli1*^+^ FL-PCs give rise to Postn^+^ CL-PCs. Nevertheless, the present contradictory results indicate a need for better clarification of 2 layers with more advanced tools such as interleaved reporter transgenic mice. It would also be interesting to study the spatial relationship among the diverse SSPCs in homeostasis and bone injury. The spatial transcriptome has already revealed the geographic distribution of various cellular components in the BM (44, 45). In the future, new spatial transcriptome techniques must be developed for hard-tissue application.

Here, we presented a comprehensive illustration of all mesenchymal clusters of the repair stage in which active fate commitment among stem cells and progenitors occurs and identified the *Postn*^+^ cluster as periosteal MPs that play a central role in bone healing. Although there is heterogeneity among *Postn*^+^ P-MPs, and this needs to be further studied, the present characterization of *Postn*^+^ P-MPs provides additional evidence for the pooling of SSCs and potential insight for the future efficient isolation of PSCs for the stem cell–based therapy of skeletal defects.

## Methods

### Sex as a biological variable.

Similar numbers of both male and female mice were used equally in all experiments and were grouped together in the analyses.

### Mice.

Postn-creER^T2^ transgenic mice were established via Biocytogen. This transgene consists of 3.9 kb of the mouse Postn promoter driving expression of the Cre (Supplemental Figure 6). Detailed information for generation of *Postn-creER^T2^* mice is in Supplemental Methods. All mice were of a C57BL/6 background, including *Postn-creER^T2^, Rosa-DTA* (The Jackson Laboratory, 009669) (46), *Gli-creER^T2^* (The Jackson Laboratory, 007913) (47), *tdTomato* (The Jackson Laboratory, 007909) (48). *CreER^T2^*-expressing mice and/or the sex-matched littermate control were i.p. injected with 70 mg/kg tamoxifen to induce the recombination of Cre. The time frame of the tamoxifen injection strategy was described in detail above. All mice were kept in a specific pathogen–free environment with temperatures of 19°C–25°C and 40%–60% humidity.

### Ablation studies.

*Postn-CreER^T2^* transgenic mice were a gift from Xianglong Han (West China Hospital of Stomatology, Sichuan University, Chengdu, China). Rosa-DTA mice were mated with *Postn-CreER^T2^; tdTomato* mice to generate *Postn-CreER^T2^; DTA; tdTomato* mice. Two-month-old *Postn-CreER^T2^; tdTomato* or *Postn-CreER^T2^; DTA; tdTomato* mice were i.p. injected with Tamoxifen (75 mg/kg) for 5 consecutive days. To study the effects of Postn^+^ cells in bone homeostasis, the femurs were analyzed 2 months after the last Tamoxifen injection starting at 2-month-old. To study the significance of the *Postn^+^* population on the bone healing, bone fracture was established. Tamoxifen was injected at 0–4 days after fracture. To evaluate the ablation efficacy, the mice were euthanized 2 days after the last Tamoxifen injection. Otherwise, the mice were harvested 14 days or 1 month after fracture for phenotypic study.

### Bone fracture, drill injury, and calvarial defect.

Mice were anesthetized with isoflurane. Before and after all the following animal operations, mice were injected with 1.2 mg/kg buprenorphine SR for pain ease.

For drill injury, the incision was made in the skin corresponding to the middle region of the femur shaft. A hole was drilled into the cortical bone to expose the BM with a dental drill of 1.6 mm in diameter. The skin is closed with a #4/0 suture.

For bone fractures, the incision was made along the longitudinal axis of the femur shaft. The femurs were cut with tissue scissors in the middle region of the longitudinal axis of the femur, and the intramedullary steel was inserted from the knee joint through the BM compartment with a 27-gauge needle and then closed with a #4/0 gauge suture. For recurring fracture, 1 month after fracture, mice were anesthetized and femurs were exposed as mentioned above in first fracture. Cuts were made in the vertical middle of callus by scissors until reaching the steel pin. At least 2 cuts were needed to disconnect the callus to establish bicortical fractures. Then, muscles and skin were sutured.

For calvarial defect, we created a circular critical size defect with a diameter of 3.5 mm in the parietal bone.

### Tissue dissociation.

To isolate the periosteal cells, the muscle was removed from the femur. The callus was separated and thoroughly cleaned to remove BM. The callus was then crushed and digested in type I collagenase (3 mg/mL, MilliporeSigma) at 37°C for 1 hour. The digested cells were then transferred to PBS, centrifuged (500*g*), and resuspended in PBS plus 2% serum. A single-cell suspension was produced by filtration through a 40 mm mesh.

### scRNA-Seq.

The sorted lineage^–^ cells from periosteal callus were captured and sequenced via 10X Genomics scRNA-Seq following the instructions of 10X Chromium Next GEM Single Cell 3′ GEM kit (10X Genomics, v.3.1). Barcoded cells were selected, and the barcoded mRNA was reverse transcribed to cDNA. The libraries were sequenced on the Illumina NovaSeq 6000 platform. Sequence data were aligned to the mouse genome (v.M20) and UMI collapsed with Cell Ranger kit (v.4.0.0, 10X Genomics). We used Seurat v4 package (v.1.8.0.) to analyze the data. For quality filtering, the multiplets and poor-quality cells that were of a gene count of fewer than 500 or more than 5,000 and of more than 10% mitochondrial and 5% hemoglobin genes were excluded. Genes expressed by fewer than 3 cells were excluded. Batch correction was realized using ComBat implementation and analyzed proportionally. Dimensionality reduction and Leiden clustering and subclustering were done by selecting parameters based on a Principal Component Analysis (PCA) elbow plot. Lineage trajectory was conducted using scVelo RNA velocity. Integration was carried out using Seurat’s Canonical Correlation Analysis (CCA) approach to correct for batch effects. Visualization of the data was achieved through nonlinear dimensionality reduction techniques such as t-SNE and Uniform Manifold Approximation and Projection (UMAP). Lineage trajectory was conducted using Monocle2 to uncover developmental pathways or progression stages. CellChat was used to infer and analyze cell-cell communication networks based on known ligand-receptor interactions.

### Immunofluorescence staining.

The mouse femurs were isolated, the skin and the muscle were removed carefully, and the samples were fixed in 4% paraformaldehyde overnight and then washed several times with PBS. Femurs were decalcified in PBS containing 14% EDTA for 1 week with constant agitation and dehydrate with 30% sucrose overnight. Bones were sliced into 7 μm–thick sections using a CryoJane Tape Transfer System (Leica). Samples were subjected to 0.5% Triton X (BioFroxx) in PBS for 10 minutes and washed with PBS before being blocked with goat serum (Invitrogen) for 30 minutes. The samples were incubated at 4°C overnight with the following antibodies: rabbit anti-Osx (1:200; 22552, Abcam), rabbit anti-Acan (1:200; AB1031, Merck), goat anti-Pdgfrα (1:100; 17-1401-81, eBioscience), rabbit anti-Ctsk (1:100; 19027, Abcam), goat anti-Ki67 (1:100; 9129, Cell Signaling Technology), goat anti-CD44 (1:100; 14-0441-82, eBioscience), goat anti-CD34 (1:100; 48740, Signalway Antibody), rabbit anti-Postn (1:100; 79946, Abcam), or goat anti-CD200 (1:100; 314662, Abcam; MA5-17980 Thermo Fisher Scientific). Then the sections were treated with the secondary antibodies anti–rabbit Alexa Fluor 488 (1:200; A11008, Thermo Fisher Scientific) at room temperature for 1 hour. The sections were then counterstained with DAPI and mounted with a fluorescence quenching–resistant mounting reagent (Invitrogen). Confocal Images were acquired by the Olympus confocal microscope, processed, and quantified using ImageJ (NIH).

### Histological study.

Safranin O Fast Green Staining was done by the Saffron O and Fast Green Stain Kit (Solarbio, G1371). H&E staining was carried out by the H&E Stain Kit (Solarbio, G1120). Masson’s trichrome staining was conducted via the Masson’s Trichrome Stain Kit (Solarbio, G1340).

### Flow cytometry.

Samples were stained for flow cytometry using a combination of the following antibodies: anti-CD31 (1:100; 25-0311-82, eBioscience), anti-CD45 (1:100; 25-0451-82, eBioscience), anti-TER119 (1:100; 25-5921-82, eBioscience). All staining was performed on ice for 0.5 hours. Identification and screening of dead cells were achieved by the 7-AAD staining. The lin^–^ live cells were selected for scRNA-Seq. To analyze the tdTomato–labeled cells, additional antibodies were used such as anti-Sca1 (17-5981-82, eBioscience), anti-Pdgfra (203491, Abcam), anti-CD200, and anti-CD105. The FACS data were acquired by FACS flow cytometer (Beckman) and analyzed by CytExpert software.

### ISH.

ISH was done using the RNAscope Multiplex Fluorescent Reagent Kit V2 (ACD Bio, 323100) combined with the RNAscope Probe-New Target Probe following the manual. All the hybridization and incubation steps were done with hybridization and incubation steps in the HybEZ Hybridization System (110V, 310010; 220V, 310013). Briefly, the frozen slides were subjected to hydrogen peroxide treatment for approximately 10 minutes and RNAscope Protease III for 15 minutes before RNAscope detection. Probe hybridization were realized at 40°C for 2 hours. The slides were then hybridized with RNAscope MultiChannel II Fluorescence AMP 1 AMP 2 and AMP 3 sequentially. The signal of the probe were then labeled with RNAscope Multichannel Fluorescence II HRP-C1 for 15 minutes and Opal 520 for 10 minutes. The counterstaining, mounting, confocal image photographing, image processing, and analysis were done as immunofluorescence staining, described above.

### μ-CT analysis.

Thirty days after fracture, femurs from mice were fixed in 4% paraformaldehyde at 4°C overnight. Samples were washed with PBS and scanned using Scanco Medical μCT. The voltage was 55 kV, and the current was 0.145 mA (mCT 35; Scanco). The threshold was set 180–1,000 to separate mineralized hard tissue from air and soft tissue. To study the callus parameters, the contour of the callus was outlined using Scanco Medical software.

### Statistics.

All experiments were conducted independently at least 3 times on different days. Mice were randomly assigned to experiments. The statistical analyses were done by standard 2-tailed Student’s *t* test. *P* values less than 0.05 were considered statistically significant. The analytical data were acquired by the GraphPad Prism v7.03. *P* values are indicated on each graph.

### Study approval.

All mouse experiments were conducted in accordance with the protocol approved by the Ethics Committee of West China School of Stomatology, Sichuan University (WCHSIRB-D-2023-173).

### Data availability.

The scRNA-Seq data produced in this study were deposited to the NCBI’s GEO database (accession no. GSE255137). Values for all data points in graphs are available in the Supporting Data Values file. Additional information is available upon request.

## Author contributions

Conceptualization was contributed by YS, LY, and BY. Analyses and interpretation of scRNA-Seq were contributed by FS, YL, and XC. Animal keeping and model establishment were contributed by QM. Supervision was contributed by LY and YS. Writing of the original draft was contributed by BY. Review and editing were contributed by BY, YS, LY, and XH.

## Supplementary Material

Supplemental data

Supporting data values

## Version 1 10/08/2024

Electronic publication
