# Supplementary material for: Identification of Postn+ periosteal progenitor cells with bone regenerative potential
Source: JCI Insight. 2024 Oct 8;9(19):e182524. doi: 10.1172/jci.insight.182524 (PMC11466188; doi:10.1172/jci.insight.182524)
Supplement: Supplemental data [file jciinsight-9-182524-s226.pdf]

Supplemental information

Supplemental data

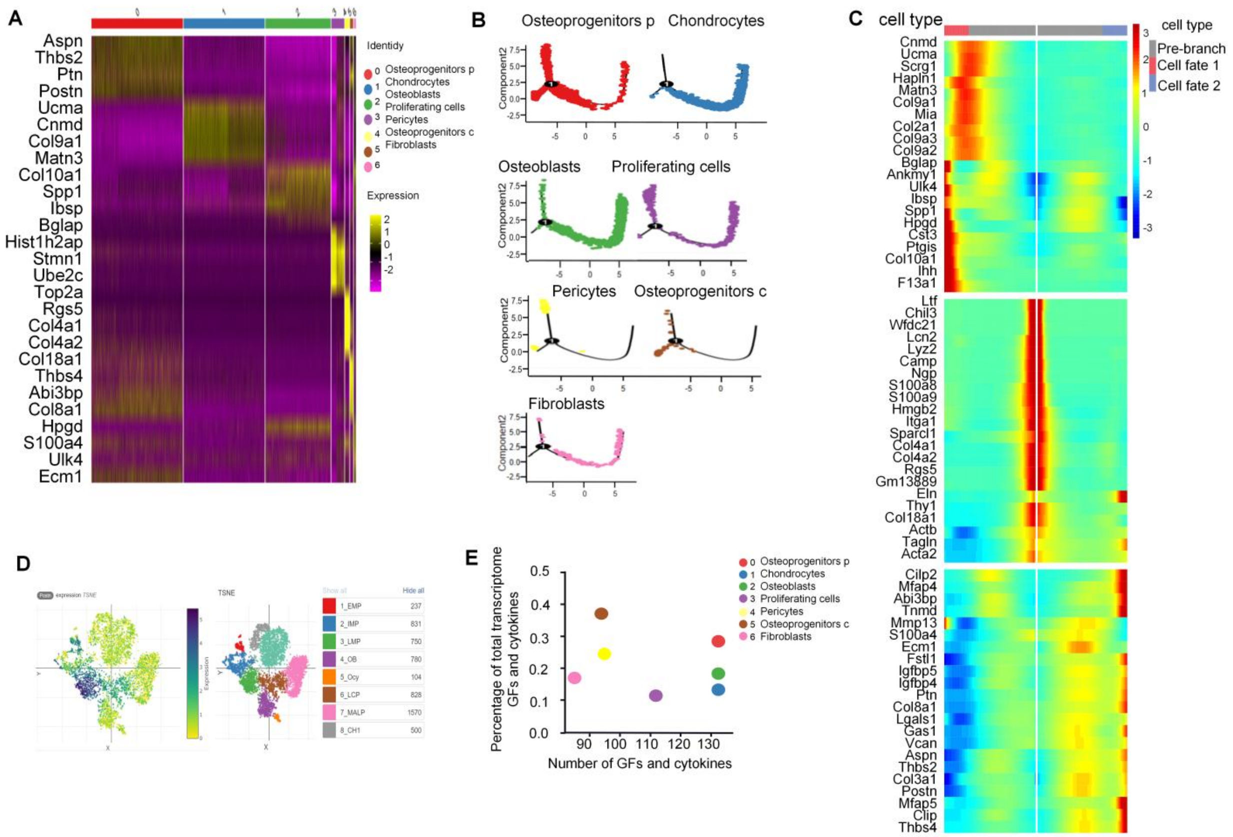

Supplemental Figure 1. Identification of *Postn*⁺ P-SSCs utilizing scRNA transcriptome profiling. (A) The heat map revealed the top 4 gene signatures in respective clusters. **(B)** Pseudotime ordering of callus mesenchymal cells of each cluster by pseudotime Monocle trajectory plot. **(C)** Heatmap of the pseudotime -dependent genes. **(D)** Distribution of *Postn* expressing cells in femoral mesenchymal clusters by TSNE. Data were produced by the dataset (GSE108892) uploaded by Leilei, et al. EMP: early mesenchymal progenitors; IMP: intermediate mesenchymal progenitors; LMP: late mesenchymal progenitors; OB: osteoblasts; Ocy: osteocytes; LCP: lineage committed progenitors; MALP: marrow adipogenic lineage precursors; CH1: chondrocytes. **(E)** Number and percentage of total transcriptome of growth factors and cytokines in each cluster.

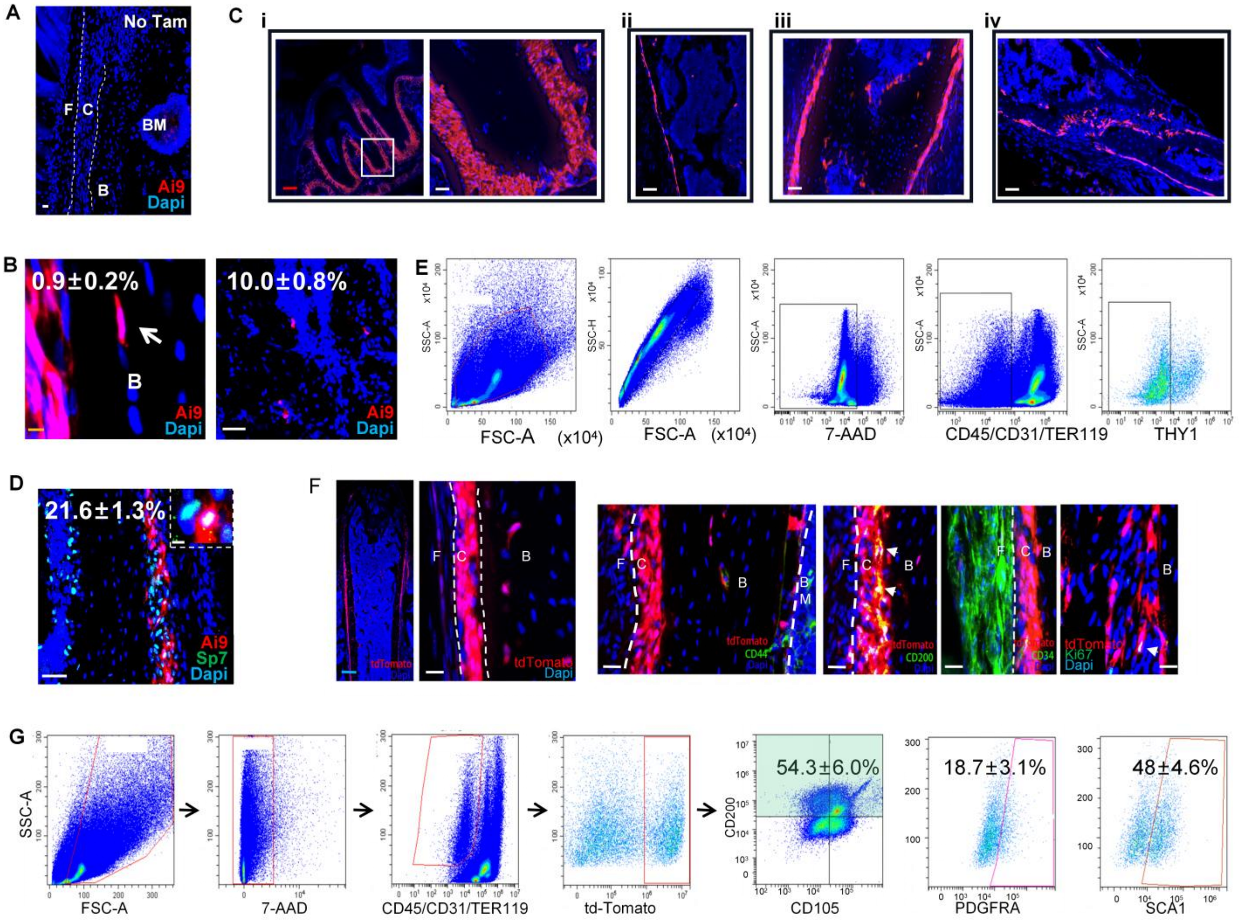

Supplemental Figure 2. *Postn* marks long-term quiescent skeletal stem cells within the periosteum. (A) Leak of *Cre* was barely observed in the absence of tamoxifen injection in *Postn-CreER^{T2}; tdTomato*. **(B)** Distribution of *tdTomato* in trabecular bone surface in cortical bone and bone marrow compartment. The arrow indicates the osteocytes in cortical bone. **(C)** *Postn* reporter activity in teeth (i), vertebra (ii), ribs (iii) and cranium (iv). **(D)** Immunofluorescence staining of Sp7 in femur of *Postn-CreER^{T2}; tdTomato* mice. **(E)**The gating of flowcytometry analysis in Fig. 2C. **(F)** The distribution of *tdTomato* and immunofluorescence staining of MSCs markers and Ki67 in the femur after tracing for 2 months. **(G)** The flowcytometry analysis of *tdTomato*$^{+}$ cells in day 10 callus of *Postn-CreER^{T2}; tdTomato* mice with tamoxifen injection for 3 days starting from the fracture day. Data were present as means ± SD. Blue scale bar: 500 µm. Red scale bar: 200 µm. White scale bar: 20 µm. "F" is abbreviated for fibrous layer, "C" is abbreviated for cambium layer, "B" is abbreviated for cortical bone, "BM" is abbreviated for bone marrow.

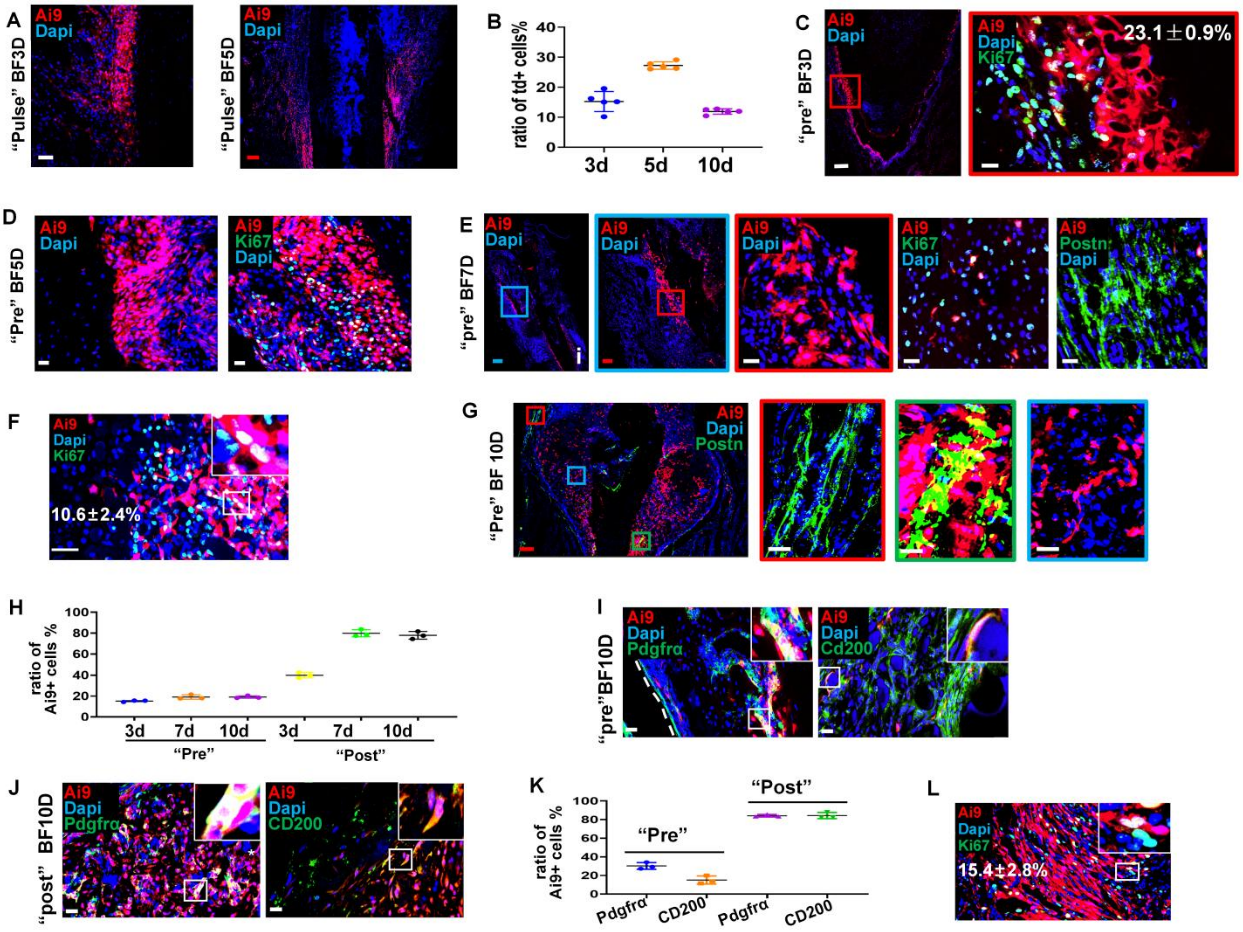

Supplemental Figure 3. ***Postn*$^{+}$ SSCs are responsible for cortical bone formation after fracture. (A)** Distribution of *Postn* expressing cells in day 3 (i) and day 5 (ii) callus. Tamoxifen was injected twice starting at 2 days before sacrifice. **(B)** Ratio of *Postn* positive cells in day 3, day 5 and day 10 callus. Tamoxifen was injected twice starting at 2 days before sacrifice. (C) Distribution of *tdTomato* and immunofluorescence staining of Ki67 in day 3 callus of *Postn-CreER^{T2}; tdTomato* mice with the "pre" strategy. " Pre" strategy means tamoxifen injection 8 days before the bone fracture for consecutive 3 days. **(D)** Distribution of *tdTomato* and immunofluorescence staining of Ki67 in day 5 callus in the "pre" strategy of *Postn-CreER^{T2}; tdTomato* mice. **(E)** Distribution of *tdTomato* and immunofluorescence staining of Ki67 and Postn in day 7 callus of *Postn-CreER^{T2}; tdTomato* mice with the "pre" strategy. **(F-G)** Immunofluorescence staining of Ki67 **(F)** and Postn **(G)** in day 10 callus of *Postn-CreER^{T2}; tdTomato* mice with the "pre" strategy. **(H)** Percentage of *tdTomato*$^{+}$ cells in day 3, day 7 and day 10 callus with the "pre" and "post" strategy (n = 3 for each group). "Pre" means tamoxifen injection 8 days before the bone fracture for consecutive 3 days. "Post" means tamoxifen injection starting at the fracture day for 3 days. **(I-K)** Immunofluorescence staining of Pdgfrα and CD200 in day 10 callus of *Postn-creER^{T2}; tdTomato* mice with "pre" **(I)** and "post" **(J)** strategy and the quantitative percentage **(K)**. **(L)** Immunofluorescence staining of Ki67 in day 10 callus of *Postn-CreERT2; tdTomato* mice with the "post" strategy (n = 3). Data were present as means ± SD. Blue scale bar: 500 μm. Red scale bar: 200 μm. White scale bar: 20 μm. Orange scale bar: 5 μm. "F" is abbreviated for fibrous layer, "C" is abbreviated for cambium layer, "B" is abbreviated for cortical bone, "E" is abbreviated for endosteum, "P" is abbreviated for periosteum.

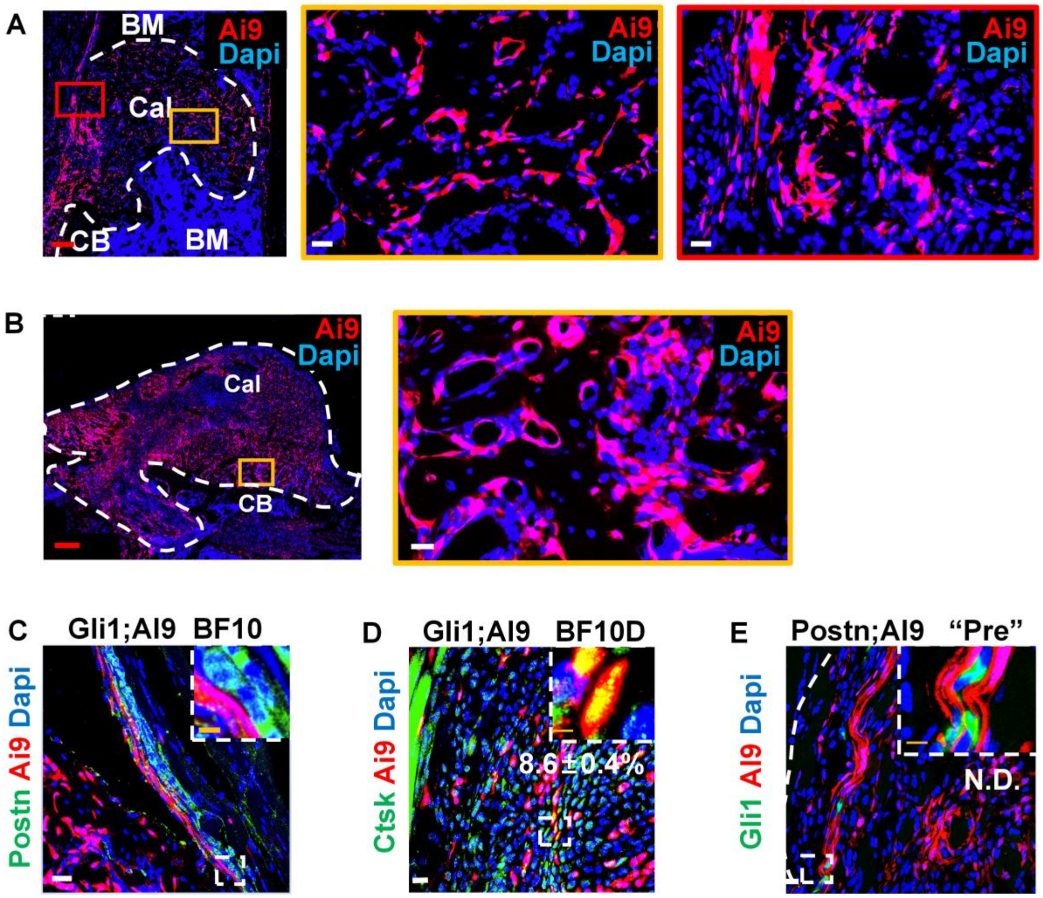

Supplemental Figure 4. ***Postn*$^+$ cells can be reactivated in successive bone injuries. (A)** Distribution of *tdTomato*$^+$ cells in day 10 callus of *Postn-creER^{T2}*; *tdTomato* mice. Drill injury was performed in the mice and tamoxifen was injected for 3 days starting from the surgery day. **(B)** Distribution of *tdTomato*$^+$ cells in *Postn-creER^{T2}*; *tdTomato* mice 10 days after cranial bone defect in *Postn-creER^{T2}; tdTomato* mice. Tamoxifen was injected for 3 days starting from the surgery day. **(C)** Immunofluorescence staining of POSTN in day 10 callus in *Gli1-creER^{T2}; tdTomato* mice. **(D)** Immunofluorescence staining of CTSK in day 10 callus in *Gli1-creER^{T2}; tdTomato* mice. **(E)** RNA scope of *Gli1* expression in day 10 callus of *Postn-creER^{T2}; tdTomato* mice. Tamoxifen was administered 8 days before bone fracture for 3 consecutive days. Data were present as mean±SD. Blue scale bar: 500 µm. Red scale bar: 200 µm. White scale bar: 20 µm. "CB" is abbreviated for cortical bone, "BM" is abbreviated for bone marrow, "Cal" is abbreviated for callus.

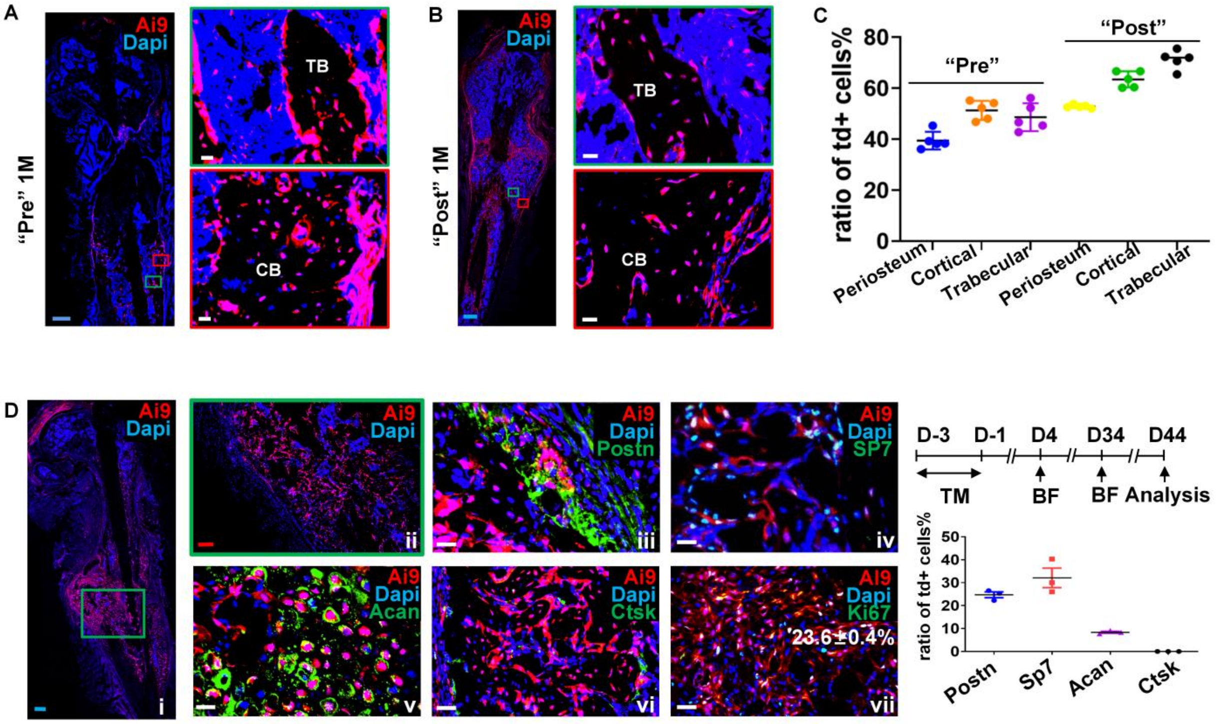

Supplemental Figure 5. *Postn*⁺ cells can be reactivated in successive bone injuries
(A) Distribution of *tdTomato*$^+$ cells one month after 1st fracture with Tamoxifen injected 8 days before 1st fracture for 3 days. **(B)** Distribution of *tdTomato*$^+$ cells one month after the first fracture with Tamoxifen injection started at the fracture day for 3 days. **(C)** Contribution of pre-existing and injury-induced *Postn*$^+$ cells to periosteum, cortical bone and trabecular bone. **(D)** Distribution of *tdTomato*$^+$ cells (i-ii) and immunofluorescence staining of POSTN (iii), SP7 (iv), ACAN (v), CTSK (vi) and KI67 (vii) in *Postn-creER*^{T2}*; tdTomato* mice 10 days after 2nd fracture. Tamoxifen was injected 8 days before 1st fracture for 3 days. Data were present as mean ± SD. Blue scale bar: 500 μm. White scale bar: 20 μm."CB" is abbreviated for cortical bone, "TB" is abbreviated for trabecular bone.

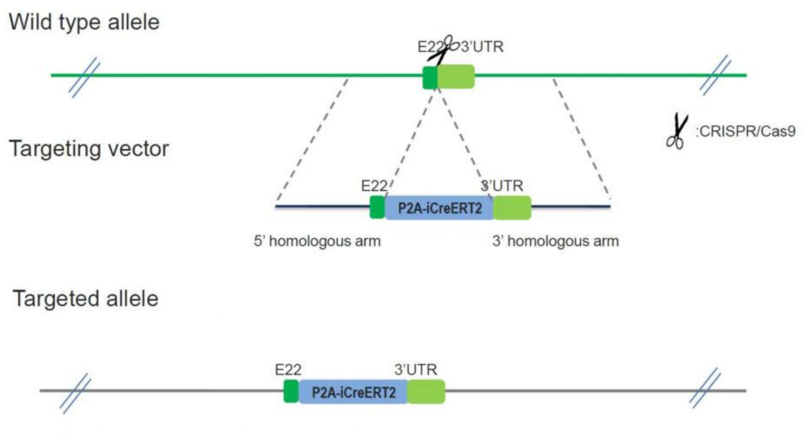

Supplemental Figure 6. **The construction strategy of *Postn-creER^{T2}* transgenic mouse.** This transgene consists of 3.9 kb of the mouse periostin promoter driving expression of the *Cre.*

Supplemental material

Generation of Postn-iCreERT2 KI mice

Screening of sgRNAs and donor vector construction

For generating Postn-iCreERT2 KI mice, we design sgRNAs targeting the sites near Stop codon. For the targeted region, CRISPR design tool (http://www.sanger.ac.uk/htgt/wge/) were used to design candidate guide RNAs. UCATM (Universal CRISPR Activity Assay) were adopted to screen the on-target activity of candidate guide RNAs.

Production of Cas9 mRNA and sgRNA

T7 RNA polymerase was used to transcribe the Cas9 mRNA and sgRNA in vitro. To generate sgRNA and Cas9 mRNA, sequence of T7 promoter was introduced to sgRNA or Cas9 mRNA template via PCR reaction. Then purify T7-Cas/sgRNA PCR products by running gel. In vitro transcription was carried out by the MEGAshortscript T7 kit (Life Technologies) following the manuals with the purified PCR products as the template. Purify Cas9 mRNA and sgRNA by MEGAclear kit and elute them by RNase-free water.

Donor vector construction

To avoid random integrations, we adopt a circular vector, which contains P2A- iCreERT2 and 2 homology arms (left 1500 bp and right1500 bp). The vector was employed as templates for repair of the Cas9/sgRNA generated DSBs. The P2A- iCreERT2 was inserted before stop codon of Postn gene.

Microinjection

C57BL/6N female mice donated the embryos and ICR mice were pseudopregnant foster mothers. Super-ovulated female C57BL/6N mice were crossed with C57BL/6N stud males. Capture the fertilized embryos in the ampullae of donor mice. Mix distinct concentrations of Cas9 mRNA and sgRNA and inject them into the cytoplasm of one-cell stage fertilized eggs. Transfer the surviving zygotes into oviducts of ICR female mice.

Southern blotting

Mice DNA was extracted and digested from tails by EcoNI (NEB) before it was separated on a 1% agarose gel. Then a nylon membrane (Hybond N+; Amersham International plc) was used to transfer the DNA. The PCR DIG probe synthesis kit (Roche Applied Science Inc.) was used to label a PCR-generated probe. Then hybridize the membrane with the probe by DIG Easy Hyb Granules (Roche Applied Science Inc.) at 42°C overnight. The hybridization signals were developed by DIG Luminescent Detection Kit (Roche Applied Science Inc.).

When labeling the probe, Taq DNA polymerase was used and DIG-11-dUTP was incorporated to generate 3′-external and internal DIG-labeled probes via PCR reaction following the manuals. Use the following primers for amplifying the 3′-external (478 bp) probe: CATTGCTGCGCGGCATCTGT (forward) and CAGAAAGCACTTTCCTGTGAGCTG (reverse). Use the following primers for the internal (529bp): CCTTCTGACTCCAATGCTGTGTC (forward) and ATCTCTGCCCAGAGTCATCCTTG (reverse).
